# Diketonylpyridinium Cations as a Support of New Ionic Liquid Crystals and Ion-Conductive Materials: Analysis of Counter-Ion Effects

**DOI:** 10.3390/ma9050360

**Published:** 2016-05-12

**Authors:** María Jesús Pastor, Cristián Cuerva, José A. Campo, Rainer Schmidt, María Rosario Torres, Mercedes Cano

**Affiliations:** 1 Departamento Química Inorgánica I, Facultad CC. Químicas, Universidad Complutense, Madrid E-28040, Spain; mjpastor@ucm.es (M.J.P.); c.cuerva@ucm.es (C.C.); jacampo@ucm.es (J.A.C.); 2 Departamento Física Aplicada III, Facultad CC. Físicas, GFMC, Universidad Complutense, Madrid E-28040, Spain; rainer.schmidt@fis.ucm.es; 3 Laboratorio de Difracción de Rayos-X, Facultad CC. Químicas, Universidad Complutense, Madrid E-28040, Spain; mrtorres@ucm.es

**Keywords:** ionic salts, liquid crystals, ionic liquid crystals, smectic mesophase, polymorphism, ionic conductivity

## Abstract

Ionic liquid crystals (ILCs) allow the combination of the high ionic conductivity of ionic liquids (ILs) with the supramolecular organization of liquid crystals (LCs). ILCs salts were obtained by the assembly of long-chained diketonylpyridinium cations of the type [HOO^R(n)pyH^]^+^ and BF_4_^−^, ReO_4_^−^, NO_3_^−^, CF_3_SO_3_^−^, CuCl_4_^2−^ counter-ions. We have studied the thermal behavior of five series of compounds by differential scanning calorimetry (DSC) and hot stage polarized light optical microscopy (POM). All materials show thermotropic mesomorphism as well as crystalline polymorphism. X-ray diffraction of the [HOO^R(12)pyH^][ReO_4_] crystal reveals a layered structure with alternating polar and apolar sublayers. The mesophases also exhibit a lamellar arrangement detected by variable temperature powder X-ray diffraction. The CuCl_4_^2−^ salts exhibit the best LC properties followed by the ReO_4_^−^ ones due to low melting temperature and wide range of existence. The conductivity was probed for the mesophases in one species each from the ReO_4_^−^, and CuCl_4_^2−^ families, and for the solid phase in one of the non-mesomorphic Cl^−^ salts. The highest ionic conductivity was found for the smectic mesophase of the ReO_4_^−^ containing salt, whereas the solid phases of all salts were dominated by electronic contributions. The ionic conductivity may be favored by the mesophase lamellar structure.

## 1. Introduction

Ionic liquids (ILs) are commonly defined as salts that are liquid at or below 100 °C. These materials exhibit useful properties such as good thermal stability, low vapor pressure, a wide liquid range and ionic conductivity [1] among others, which are of particular interest for various applications [2]. For example, ILs have been applied as alternative solvents in green chemistry for many decades now due to their low volatility avoiding basic contamination. In addition, applications involving biphasic catalysis have been extensively investigated [3,4].

Ionic liquid crystals (ILCs) can be defined as materials in which properties of ILs are combined with those of liquid crystals (LCs) [5]. In particular, the ionic conductivity of ILs can be combined with the characteristic anisotropy of the LCs [6], which results in the interesting possibility of achieving ion-conductive pathways through which ions may be transported in the selected directions of the LC arrangements. This strategy seems promising for the development of possibly a wide range of ion-conductive LCs. Therefore, some examples are the imidazolium-type ionic liquid crystals as effective electrolytes for dye-sensitized TiO_2_ solar cells [7], or the 2D ion-conductive ILCs obtained by co-assembly of imidazolium salts and calamitic LCs, which have been proved useful as ion-conductive media for lithium salts [8]. There are other examples of ionic liquid crystals described in the literature, which should be ion-conductive materials by their ionic nature [9,10,11,12,13,14,15,16,17,18,19,20,21].

For designing thermotropic ILCs, it is important to first consider that the thermal behavior may depend on the shape of the cations and anions forming those materials as well as the interionic interactions. The thermotropic mesomorphic behavior of ILCs has been reported before for several ionic compounds containing anisotropic rod-like cations as mesogenic unities and classic inorganic or organic anions. Typical anions are simple halides or pseudo spherical anions like BF_4_^−^, PF_6_^−^ or ClO_4_^−^ [22,23,24], or some others with lower symmetry like CF_3_SO_3_^−^. Some of the cations reported previously are long-chained substituted triazolium [25,26], pyridinium [27,28], or piperidinium [29] among others, but the most extensive studies deal with the salts of the imidazolium cations [30,31,32].

In addition, anionic metal complexes of the type [MCl_4_]^2−^ (M = Cd, Zn) were also proved useful as counter-ions to achieve ILCs with alkylpyridinium cations giving rise to lamellar mesophases [33]. In particular, in [PdX_4_]^2−^ derivatives the LC temperature decreases by changing the halide (X) from Cl^−^ to I^−^. This trend is related to the decreasing strength of the H-bonding interaction between the [PdX_4_]^2−^ and the pyridinium cations (Cl > Br > I) [34].

Taking into account all the previous results in ILCs, it seems clear that the observed mesomorphism can depend on different factors like the chain length of the cation [35,36,37], the nature of the anion [38], the H-bonding and/or interionic interactions [39].

In previous works of our group, we described ILCs based on pyrazolium salts of the type [H_2_pz^R(n)^][A] (R(n) = C_6_H_4_OC_n_H_2n+1_, *n* = 8, 10, 12, 14, 16, 18; A = Cl^−^, BF_4_^−^, ReO_4_^−^, SbF_6_^−^, CF_3_SO_3_^−^ (OTf), CH_3_-*p*-C_6_H_4_SO_3_^−^), which exhibited smectic mesophases (SmA) with the exception of the derivatives with the bulkiest CF_3_SO_3_^−^ (OTf) and CH_3_-*p*-C_6_H_4_SO_3_^−^ counter-ions [40]. Moreover, we reported studies of ILCs based on pyridinium-containing diketone cations of the type [HOO^R(n)pyH^]^+^ (R(n) = C_6_H_4_OC_n_H_2n+1_, *n* = 10, 12, 14, 16, 18) which were isolated with [ZnCl_4_]^2−^ and Cl^−^ counter-ions [41]. In that work we reported on remarkable mesomorphic behavior of the Zn derivatives exhibiting enantiotropic mesophases with an extensive range of stability, in contrast to its absence on the chlorides ones.

To optimize the practical performance of ILCs, detailed knowledge about their physical and chemical properties is required and in particular, the evaluation of the typical phase transitions appears to be determinant.

In this work, we examine the LC behavior of novel [HOO^R(n)pyH^]_m_[A] (*n* = 12, 14, 16, 18 and *m* = 1: A = BF_4_^−^, ReO_4_^−^, CF_3_SO_3_^−^, NO_3_^−^; *n* = 12, 18 and *m* = 2: A = CuCl_4_^2−^) salts. We propose that the presence of tetrahedral or more planar anions help achieving layered smectic mesophases, because these anions were proved to favor a layer type structure. The influence of the LC organization of the cations will be examined and related to the conducting properties of the entire compounds.

## 2. Results and Discussion

### 2.1. Synthesis and Characterization

**Scheme 1:**
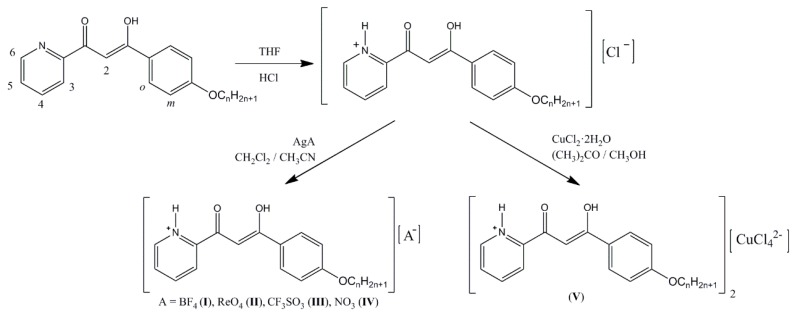
Synthetic route to obtain the ionic salts. The numbering scheme used in the ^1^H-NMR assignments is also denoted.

Five new families of ionic compounds containing diketonylpyridinium cations and different counter-ions of the type [HOO^R(n)pyH^]_m_[A] (*n* = 12, 14, 16, 18 and *m* = 1: A = BF_4_^−^, ReO_4_^−^, CF_3_SO_3_^−^, NO_3_^−^; *n* = 12, 18 and *m* = 2: A = CuCl_4_^2−^) (Table 1, Scheme 1) have been prepared and their thermal behavior and ionic conductivity were studied. The synthesis was carried out by reaction of the silver (AgA; A = BF_4_^−^, NO_3_^−^, CF_3_SO_3_^−^, ReO_4_^−^) or copper (CuCl_2_·2H_2_O) salts and the corresponding ß-diketonylpyridinium chloride in a 1:1 and 1:2 molar ratio, respectively (see Scheme 1). The salts **1**–**16** were isolated as yellow solids and **17** and **18** as green solids, which were all soluble in polar solvents. Elemental analyses and IR spectroscopy of all compounds, and ^1^H-NMR of salts **1**–**16**, were used to characterize and establish their identity. In the particular case of the compound **18**, MALDI-TOF mass spectrometry was carried out as well.

The IR spectra of all compounds exhibit a *ν*(CO) absorption band at *ca.* 1602 cm^−1^, which was not modified with respect to that of the starting chloride derivatives. The slight differences observed between the families related to the broadness and/or structuration are probably associated to the different structural features and therefore attributed to solid effects. A strong band observed at *ca*. 785 cm^−1^ is characteristic of the *γ*(CH) vibration of the pyridyl group. In addition, bands at *ca.* 1256–1084, 1400, 1056 and 1032, 912 cm^−1^ were assigned to *ν*(BF), *ν*(NO), *ν*(SO) and *ν*(ReO) of the corresponding counter-ions, BF_4_^−^, NO_3_^−^, CF_3_SO_3_^−^, ReO_4_^−^, respectively (Section 3.3). These values are in agreement with their ionic nature in the new compound [42].

The ^1^H-NMR spectra of the compounds [HOO^R(n)pyH^][A] (**1**–**16**) in (CD_3_)_2_CO at room temperature display the characteristic signals of the pyridyl, alkyloxyphenyl and *β*-diketone core protons. Concerning the pyridyl group, all compounds of the families **I**–**III** exhibit the H3, H4, H5 and H6 proton signals down-field shifted with respect to those of the chloride salts. In contrast, in the nitrate derivatives (**IV**), these signals appear up-field shifted. On the other hand, the phenyl group signals were not modified related to the starting chloride derivatives. For all compounds the presence of duplicated signals of almost all protons are in agreement with the presence of the two tautomeric enol and keto forms of the cation in solution. In all the cases the enolic form was the predominant one as can be determined from the signal intensity ratio of the CH/CH_2_ protons. Therefore, a singlet signal of the proton attached to the carbon C(2), observed at *ca.* 7.63 ppm is due to the enol form while the keto tautomer is confirmed by the weak singlet at *ca*. 4.80 ppm of the central C(2)H_2_ group of the diketone core.

In order to determine the molecular formula of paramagnetic compounds of the type **V**, MALDI-TOF mass spectrometry was carried out for compound **18** as a representative example from this family. The spectrum shows the molecular ion M^+^ at *m*/*z* 1210, which is consistent with the formula [HOO^R(18)pyH^]_2_[CuCl_4_]·H_2_O proposed from elemental analysis. Additionally, a peak at *m*/*z* 495 corresponds to the fragment [HOO^R(18)pyH^]^+^.

### 2.2. X-ray Crystal Structure of [HOO^R(12)pyH^][ReO_4_] (**5**)

We have attempted to grow single crystals of the salts of different families, but only [HOO^R(12)pyH^][ReO_4_] (**5**) gave crystals with sufficient crystalline quality for X-ray analysis. In the same and related series it appeared to be more and more difficult to obtain good crystalline quality with the chain length increasing. We have resolved only the X-ray structure of **5** but the low quality of the crystal data implies that the extracted crystal parameters may be affected by a perceptible fitting error. Nevertheless, the X-ray data turned out to be useful to correlate the detected molecular organization and crystal packing with the chemical and physical properties as described below.

**Figure 1:**
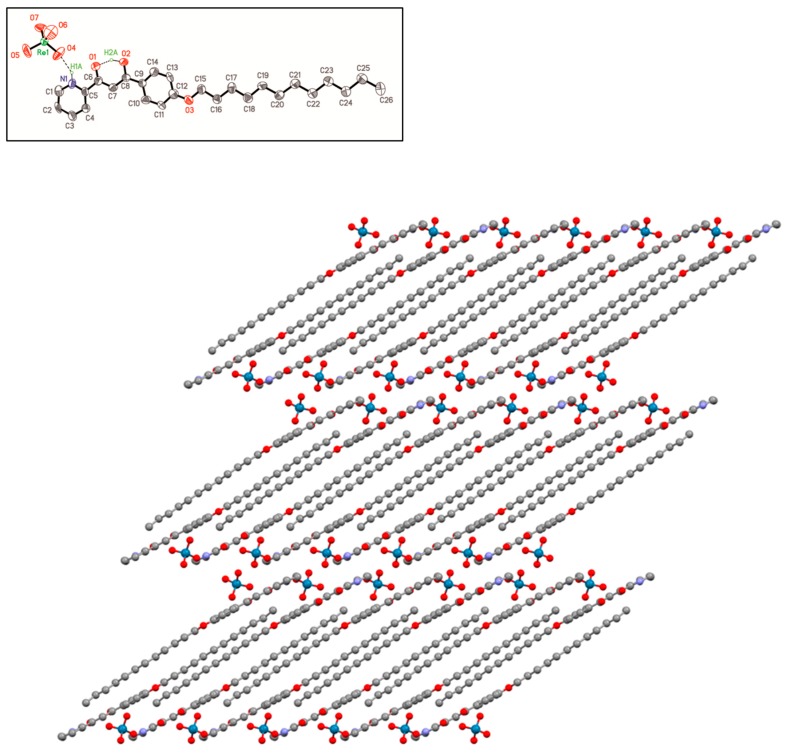
Crystal structure of [HOO^R(12)pyH^][ReO_4_] (**5**): Layer-type distribution showing interdigitated chains. The inset shows the ORTEP plot with 40% probability.

Suitable yellow colored crystals of [HOO^R(12)pyH^][ReO_4_] had been obtained from a CH_2_Cl_2_/CH_3_CN solution. The compound crystallizes in the monoclinic space group *P*2_1_/*c*. The molecular structure is depicted as an inset in Figure 1 (also in Figure S1), and Tables S1 and S2 list bond distances and angles and crystal and refinement data, respectively.

The asymmetric unit consists of a pyridinium cation and a ReO_4_^−^ anion, which are linked by a N1 ··· O4 hydrogen-bond (Table S3). The most important feature of the cation is its high planarity, as it can be deduced from the dihedral angles between the diketonyl plane and phenyl and pyridine rings (values ranging between 2.8(1)° and 3.9(1)°). The hydrocarbon chain is running almost parallel to the central core (the angles between the plane normal and the line defined by the O3–C26 atoms is *ca.* 85°).

The crystal structure consists of discrete cations and anions that are organized in a lamellar fashion, where each layer exhibits alternating hydrophobic and hydrophilic regions along one crystal direction.

On a supramolecular level, the structure may be defined as an interdigitated layered structure generated by weak C–H···O hydrogen-bonds involving the counter-ions with neighboring cations (Table S3). By considering the tilting of the alkyl chains relative to the layer normal we can estimate the layer thickness to be *ca.* 17 Å. Alternatively, the structure may also be described by alternating polar and apolar organic sublayers showing a high degree of interdigitation (Figure 1 and Figure S2).

### 2.3. Thermal Behavior

The thermal phase behavior of the five series of the salts (families **I**–**V**; compounds **1**–**18**) was investigated by polarized light optical microscopy (POM), differential scanning calorimetry (DSC) and temperature dependent powder X-ray diffraction (XRD). The transitions temperatures and phase assignments are reported in Table 2.

All compounds listed in Table 2 show a LC behavior exhibiting a single enantiotropic mesomorphism that is present over a certain temperature range, which markedly depends on the counter-ion. In addition, for salts **I**–**IV** a rich crystalline polymorphism was also observed.

**Figure 2:**
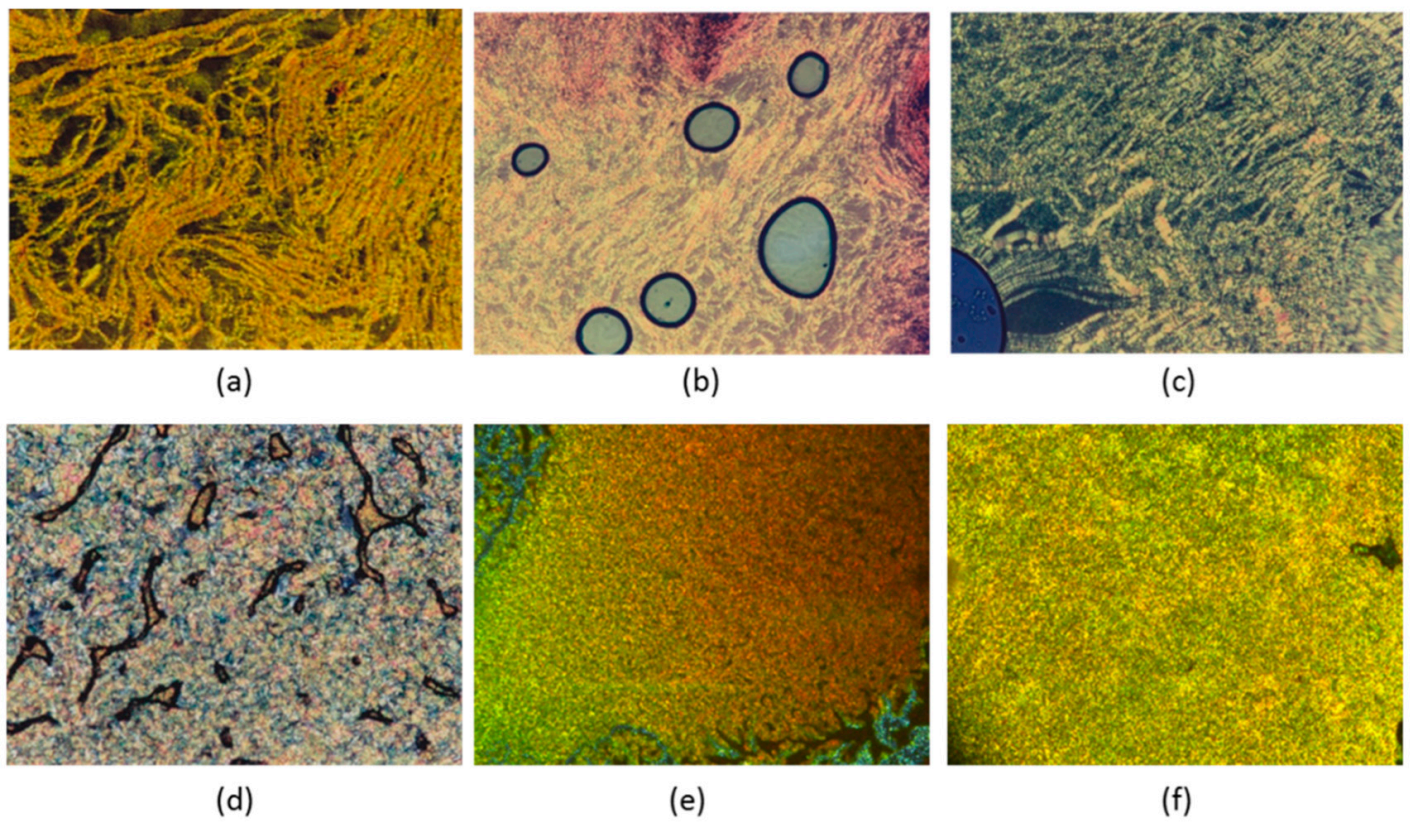
POM photomicrographs of: (**a**) **BF_4_-12** at 114 °C on cooling; (**b**) **ReO_4_-12** at 104 °C on cooling; (**c**) **CF_3_SO_3_-14** at 148 °C on cooling; (**d**) **NO_3_-12** at 112 °C on cooling; (**e**) **CuCl_4_-12** at 158 °C on heating; (**f**) **CuCl_4_-18** at 111 °C on cooling.

In light of the evidence of the POM observations, the liquid crystal phases of compounds **1**–**16** were identified as SmA mesophases on the basis of the appearance of an oily-streak texture (Figure 2a–d and Figure S3), which could be observed best upon cooling. In contrast, a “sandy” texture of a SmC mesophase was observed for compounds **17** and **18** (family **V**) (Figure 2e,f) during heating and cooling cycles. One problem with the POM observations of the mesophase, with the exception of the triflate salts, was the fact that the derivatives showed decomposition at temperatures shortly above the clearing (SmA-Iso transition). The POM observations mentioned above were therefore carried out by heating the hot-plate close to the clearing temperature and then inserting the sample. Immediately the sample was cooled to avoid decomposition and the respective texture could be observed clearly.

Generally, the DSC traces were consistent with the optical experiments (Figures S4–S10). On the first heating, the DSC thermograms of the compounds **1**–**4** (**BF_4_-n**) exhibit only one peak at *ca.* 130 °C related to the Cr-SmA transition whereas the isotropization could only be detected by POM. In the case of compounds **5**–**8** (**ReO_4_-n**), the thermograms exhibit two endothermic peaks corresponding to the solid-mesophase and the mesophase-isotropic liquid phase transitions. Additionally, several others peaks attributed to phase transitions in the solid state are also observed before the melting temperature. In the particular case of **4** and **6**, solid-solid and solid-mesophase and solid-solid processes overlap. This fact can be confirmed by the presence of two or three broad unseparated peaks in the DSC thermograms, which give rise to a high ΔH value (Table 2). However, the optical observations can confirm only the last two transitions, which attributed to the formation of a viscous birefringent SmA phase followed by clearing to an isotropic liquid. In agreement with the POM observations mentioned above, partial degradation occurs just before the isotropization, which precludes the detection of the peak corresponding to the mesophase on cooling.

The DSC traces of triflate compounds **9**–**12** (**CF_3_SO_3_-n**) show several peaks on heating. For all salts the two first peaks are related to Cr-Cr′ and Cr′-Cr″ transitions, which were not revealed by POM. A broad peak which involves two overlapped solid-mesophase and mesophase-isotrope transitions was observed for **CF_3_SO_3_-12** and **CF_3_SO_3_-14** salts. In contrast, two separated peaks corresponding to both processes were observed for **CF_3_SO_3_-16** and **CF_3_SO_3_-18** derivatives. On cooling, three exothermic peaks from isotrope-mesophase, mesophase-solid and solid-solid phase transitions can be observed, due to the higher stability of these salts toward decomposition.

The nitrate salts **13**–**16** (**NO_3_-n**) have a simpler DSC thermogram exhibiting a broad endothermic peak on heating, which involves two overlapping solid-mesophase and mesophase-isotrope transitions. This indicates a rather small existence range of the mesophase. The degradation of the sample observed by POM after the clearing temperature is evident from the presence of an exothermic peak around 145 °C (ΔH = −206.0 kJ·mol^−1^). As a consequence, the formation of the mesophase from the isotropic liquid could not be registered on cooling as mentioned above.

On the other hand, the DSC traces of the tetrachlorocuprate derivatives **17** and **18** (**CuCl_4_-n**) confirmed the mesophase formation at lower temperatures than those of the previous mentioned salts (**1**–**16**). The compound **17** shows two endothermic peaks on heating, which correspond to the solid-mesophase and the mesophase-isotropic liquid phase transitions. On the other hand, in **18** an additional exothermic peak appears after that of the solid-mesophase transition, which points towards a rather surprising crystallization of the mesophase, this fact being confirmed by the observation by POM. Finally, another endothermic peak at temperatures close to the clearing was observed, which was again related to the formation of a new mesophase which evolves to the isotropic liquid as reflected by the last endothermic peak observed in the DSC trace (Table 2). In both compounds, an extensive decomposition is produced as detected by POM, and therefore the enthalpies have higher values than the expected.

By comparing the mesomorphism of the five series of salts, we recognize a major difference in the [CuCl_4_]^2−^ derivatives (family **V**), which display a smectic C mesophases at relatively low temperature, whereas the remaining salts (**I**–**IV**) exhibit SmA mesophases at higher temperatures. In addition, the melting transitions for the [CuCl_4_]^2−^ derivatives show the highest enthalpy with respect to the remaining derivatives.

On the other hand, the series containing the tetrahedral anions ReO_4_^−^ and BF_4_^−^ have a similar behavior exhibiting melting temperatures both near 130 °C, as well as a mesophase range of *ca*. 44 °C. In contrast, in CF_3_SO_3_^−^ containing salts, the melting temperatures are increased to *ca.* 150 °C and the mesophase range decreases, being almost inappreciable in some cases. The mesomorphism of NO_3_^−^ salts follows way mirror to that of the CF_3_SO_3_^−^ derivatives, exhibiting mesophases shifted to lower temperature (*ca.* 135 °C) overlapping with the isotropization in all cases.

As a general result, it was observed that the single mesophase of the [HOO^R(n)pyH^][A] (**I**–**IV**) (*n* = 12, 14, 16, 18) derivatives appears at temperatures barely depending on “*n*”.

On the other hand, comparing the thermal behavior of [HOO^R(n)pyH^]_2_[CuCl_4_] (*n* = 12, 18) with that of the related [HOO^R(n)pyH^]_2_[ZnCl_4_] [29], it was observed that the latter salts show better thermotropic LC properties. Their mesomorphism is related to lamellar SmA mesophase, which span through a wider temperature range. The different thermal behavior of the compounds [ZnCl_4_]^2−^ and [CuCl_4_]^2−^ derivatives was clearly related to the type of the anion tetrahalometallate complex which control the mesophase nature. In both cases, although structural characteristic should be similar, given the very subtle differences between the two tetrahedral anions, the Zn derivatives exhibit Sm_O_ and SmA mesophases, while for the copper ones only SmC mesophase was observed. 

**Figure 3:**
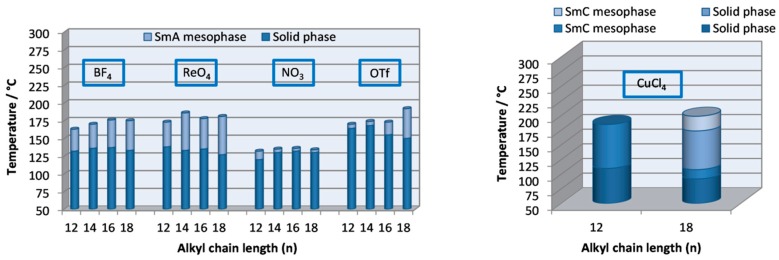
Phase transition temperatures of the different salts as dependent on the anion and the cation length.

For comparative purposes, Figure 3 despites the melting and clearing temperatures of all compounds with a similar 1:1 and 1:2 stereochemistry (**I**–**IV**).

### 2.4. Variable Temperature Powder X-ray Diffraction Studies

**Figure 4:**
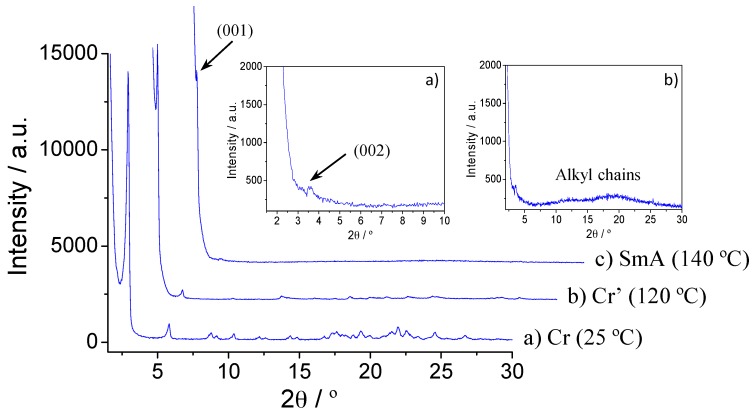
X-ray diffractograms of **ReO_4_-16** (**7**) at (**a**) 25 °C; (**b**) 120 °C and (**c**) 140 °C on heating. The insets show a magnification of the XRD trace at the mesophase temperature (**a**) showing the (002) peak and (**b**) the broad halo corresponding to the liquid-like order of the molten alkyl chains.

In order to confirm and compare the lamellar structure of the mesophases with that of the starting solids, we have studied the X-ray powder diffraction patterns of one salt from each family (**3**, **7**, **12**, **13** and **18**) at variable temperature. As a representative example, the X-ray diffraction patterns for [HOO^R(16)pyH^][ReO_4_] (**7**) at selected temperatures is presented in Figure 4.

**Figure 5:**
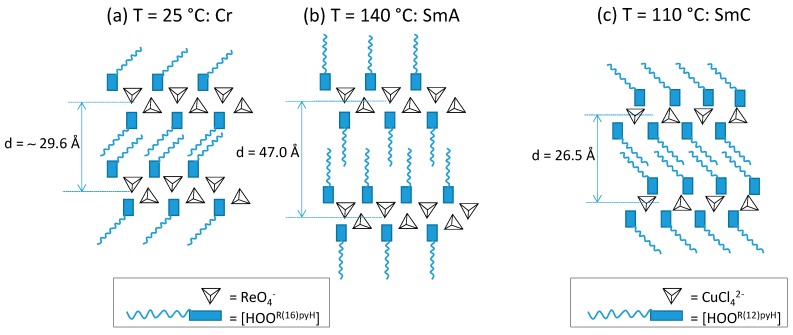
Schematic representation of the structural features: (**a**) [HOO^R(16)pyH^][ReO_4_] at the solid state based on the structure of **ReO_4_-12**; (**b**) proposal for [HOO^R(16)pyH^][ReO_4_] at the SmA mesophase; and (**c**) proposal for [HOO^R(12)pyH^]_2_[CuCl_4_] at the SmC mesophase.

At room temperature (Figure 4a), the diffraction pattern agrees with the lamellar structure of the solid in accordance with a strong peak in the low-angle region at 2*θ* = 2.98° (*d* = 29.6 Å) followed by several peaks in the medium and wide angle regions with the low intensity characteristic of a solid phase. This was stable upon heating up to 111 °C, where a solid-solid phase transition (Cr-Cr′) occurs (Figure 4a,b) that can be regarded a polymorphism. By further increasing the temperature to 135 °C the derivative enters into the mesophase, and the diffractogram collected at *ca.* 140 °C shows two peaks in the low-angle region involving the (001) and (002) reflections of the smectic mesophase (Figure 4c). These reflections confirm the lamellar arrangement of the mesophase with a layer spacing *d* = 47.0 Å, which is significantly larger as compared to the solid (Figure 5). Additionally, the broad halo, which corresponds to the liquid-like order of the molten alkyl chains appears around 5.2 Å. A further temperature increase leads to the formation of the isotropic liquid above 150 °C.

In the remaining salts, except in the NO_3_^−^ derivatives, the X-ray studies again indicated that more than one crystalline polymorph is present, in agreement with the thermal study shown above (Section 2.3).

Interestingly, the solid-solid phase transition enthalpies of the two polymorphs for the ReO_4_^−^ derivatives were much greater than those of the other salts, indicating a more dramatic structural change, probably associated with a modification of the interdigitation or possibly to a change towards a crystalline structure related to that of the SmA mesophase.

For the remaining compounds, the diffraction patterns show equivalent features. Table 3 displays the diffraction data of compounds **3**, **7**, **12**, **13**, **18** at their corresponding mesophase temperatures. Smectic mesophases were identified for all of them on the basis of the two or three peaks in the low angle region with a ratio of the *d*-spacing of 1:2 (001, 002 reflections) or 1:2:3 (001, 002, 003 reflections). In all cases, a broad halo at *ca.* 5.2 Å attributed to the liquid-like order of the molten alkyl chains was also observed.

It is interesting to note that the highest *d*-spacing (47.0 Å) was formed for the ReO_4_^−^ salt (**7**) in agreement with the bulkiest size of the counter-ion as compared to the BF_4_^−^ derivative (**3**). Furthermore, this value (*d* ≈ 47.0 Å) was also increased with respect to that found for the salt (*d* ≈ 29.6 Å) in the solid state, which is a consequence of the change of the lamellar organization from the solid to the SmA mesophase (Figure 5a,b).

On the other hand, the tetrahedral CuCl_4_^2−^ counter-ion (**17**) gives rise to a smaller *d*-spacing which could be explained by its smaller size as well as the tilted organization of cations at the SmC mesophase (Figure 5c).

### 2.5. Conductivity and Dielectric Properties

Since our ILCs may be of interest due to their potential ionic conductivity typical for ILs, we have carried out ionic conductivity measurements in order to acquire a better understanding of this physical property.

We have carried out a study of the conductivity over several selected salts from the samples investigated here, [HOO^R(12)pyH^][ReO_4_] and [HOO^R(12)pyH^]_2_[CuCl_4_] salts of the families **II** and **V** respectively, which have been shown to display the best LC behavior in terms of a low temperature solid-mesophase transition and a wide mesophase range. Furthermore, we have studied a control sample of a non mesomorphic salt ([HOO^R(12)pyH^][Cl]) that has been described in a previous work [41].

**Figure 6:**
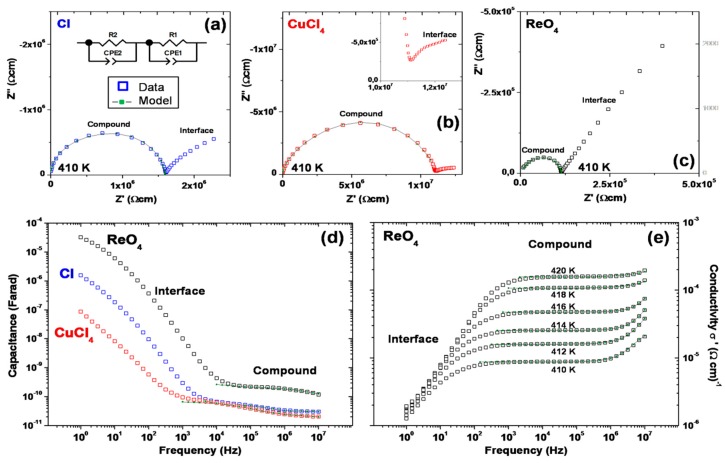
(**a**–**c**) *Z″* *vs.* *Z′* and (**d**) *C′* *vs.* *f* plots for the Cl^−^, CuCl_4_^2−^ and ReO_4_^−^ salts; (**e**) *σ′* *vs.* *f* plots for the favorable ReO_4_^−^ salt.

Figure 6a–c displays impedance plane plots of imaginary *vs.* real part of the impedance -*Z″* *vs.* *Z′*, obtained from the **Cl-12**, **CuCl_4_-12** and **ReO_4_-12** salts at 410 K (137 °C) in the solid (Cl^−^) or the smectic mesophase (CuCl_4_^2−^ and ReO_4_^−^). The semicircle of each compound represents the dielectric contribution from the ionic salts (marked as “Compound”), where the semicircle diameter corresponds to the resistivity *ρ* of the charge transport or the inverse of the long range conductivity *σ*_dc_ (*ρ* = 1/*σ*_dc_) within the compound. An additional dielectric contribution is evident at low frequency (*f)* in form of an additional semicircle or a pike-like shape, marked as “Interface” in the figure. In the case of the ReO_4_^−^ compound the pike-structure is most clearly developed, which is a hallmark feature of ionic conductivity [43,44,45]. The pike-structure occurs due to the blocking of the ionic charge carriers at the interface between the ionic salt and the electrodes. This is due to the metallic character of the electrodes, which do not possess the infrastructure for the ionic charge carriers to move or even to enter across the interface. In the Cl^−^ derivative the interface contribution resembles more a semicircle, which may imply that an electronic contribution to the charge transport may exist, whereas the ReO_4_^−^ compound undoubtedly displays the signs of ionic conductivity in form of a large pike. The inset in Figure 6b shows a magnification of the data collected at low *f* from the CuCl_4_^2−^ compound to demonstrate the pike-like feature in detail, but it is less clearly developed as compared to the ReO_4_^−^ salt and a small electronic contribution cannot be fully excluded.

In the Figure 6a panel the standard equivalent circuit model of two non-ideal resistor-capacitor (RC) elements is shown [46], which was used to fit the semicircular dielectric data at intermediate and high *f*. The semicircles displayed in Figure 6a–c are slightly non-symmetric and could be fitted adequately only with two series RC elements. The pike-like feature cannot be fitted with standard RC elements and is not accounted for in the circuit model. The ideal capacitors in the standard RC elements had been replaced by a constant phase element (CPE), which accounts for the non-ideality of the respective dielectric contribution. On a microscopic level the CPE behavior is usually explained in the framework of a jump-relaxation of the ionic carriers at high *f* [47], or alternatively by a broadening of the distribution of characteristic dielectric relaxation times *τ* across the sample [43,48]. The use of two R-CPE elements in series in the equivalent circuit instead of only one element can be justified by the occurrence of two approximately *f*-independent plateaus at intermediate and high *f* (“Compound”) in the plots of capacitance *vs.* *f* shown in Figure 6d. The strong increase of capacitance at low *f* is again a typical feature of ionic conductivity and can be associated with the “Interface”.

Further insights into the ionic conduction mechanism can be obtained by plotting the data in form of the real part of conductivity *σ*′ *vs.* *f*. In Figure 6e the data obtained from the ReO_4_^−^ compound at selected temperatures in the smectic mesophase show the following features: A thermally Arrhenius activated plateau at intermediate *f* corresponds to the long range conductivity *σ*′_dc_ within the liquid ionic salt (“Compound”), whereas a small dispersion at higher *f*, especially at lower *T*, may be associated with a certain distribution of relaxation times *τ* [49]. The alternative explanation of a possible Jonscher-type universal response law could not be verified due to limited *f*-resolution. For the Jonscher-type universal dielectric response the high-*f* data points for different *T* would be expected to fall onto the same curve [50], which is not what we observe. Still, the occurrence of possible Jonscher type dielectric response at higher *f* beyond the experimental limits cannot be excluded. Again, at low *f* the contribution from the Interface is displayed.

**Figure 7:**
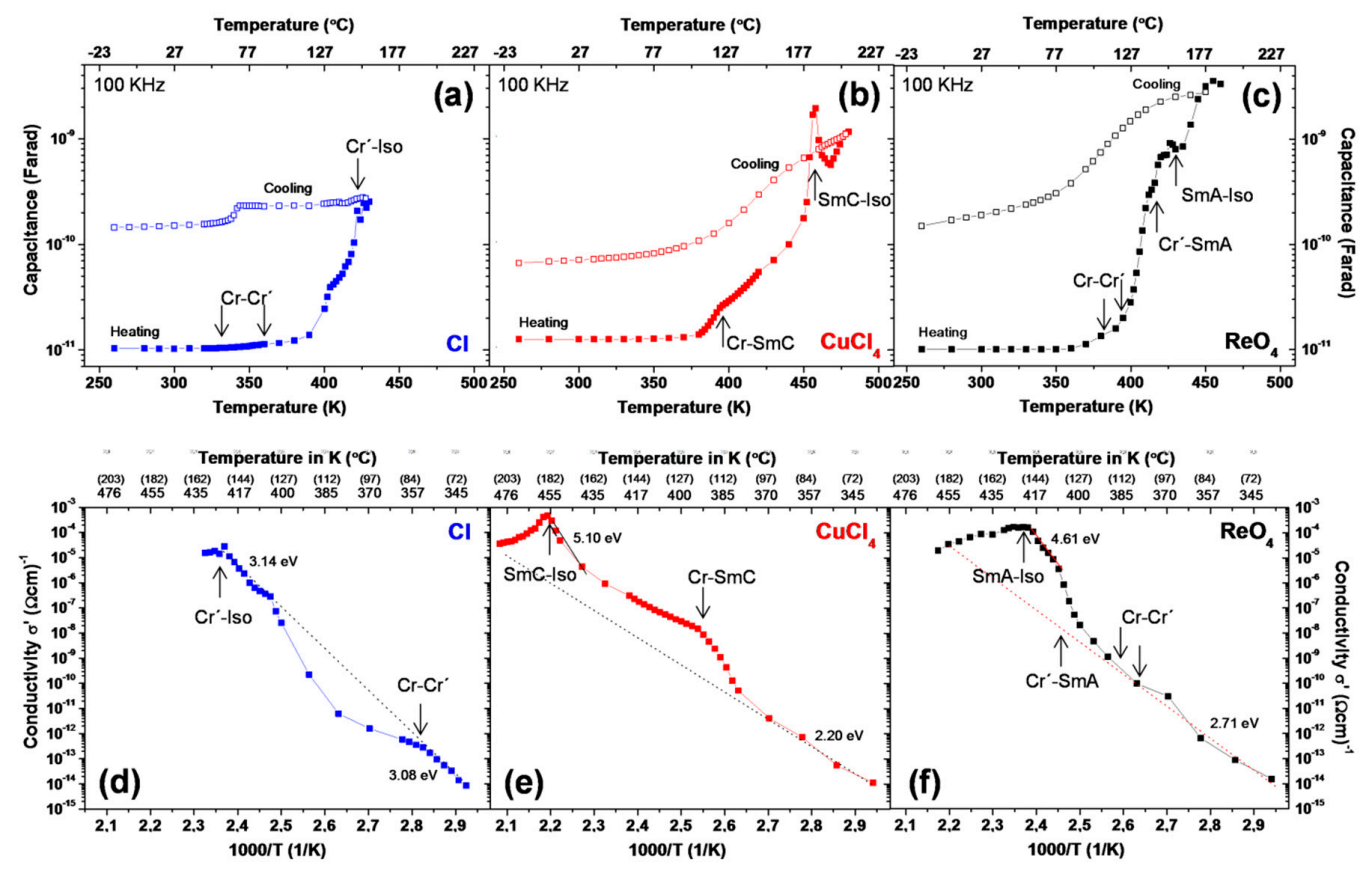
(**a**–**c**) *C′* *vs.* *T* and (**d**–**f**) *σ*′ *vs.* *f* plots for the Cl^−^, CuCl_4_^2−^ and ReO_4_^−^- based compounds.

The *T*-dependences of the capacitance for the compounds investigated are summarized over the full *T*-range accessible in Figure 7a–c. The different phase transitions in the three compounds investigated are reflected in the curves to a different extent. Whereas some solid-solid transitions (Cr-Cr′) are hardly visible at all, the solid-mesophase (Cr-Sm) and the mesophase-to-liquid (Sm-Iso) transitions are displayed clearly as a sharp increase of capacitance with increasing temperature or a peak-like structure in these curves. The compounds were found to all decompose shortly above the Cr-Iso (Cl^−^) or the Sm-Iso transitions (CuCl_4_^2−^ and ReO_4_^−^), and consequently irreversibility is indicated in the cooling-curves of capacitance *vs.* *T*. The long range conductivity or *σ*_dc_ values extracted from the equivalent circuit fits for the compounds studied are shown in plots of *σ*_dc_ *vs.* 1/*T* in Figure 7d–f. The data displayed were recorded upon heating, where the phase transitions are again displayed to different extents.

The Cl^−^ compound exhibits two approximately linear regimes that allow extracting the activation energy *E*_A_ from the respective Arrhenius plots. In the absence of a smectic mesophase in the Cl^−^ compound similar *E*_A_ values (probably the same within experimental error) are encountered for the low and high *T* ranges. The two regimes are separated by transitional features that may be associated with the solid-solid (Cr-Cr′) phase transition. In fact, the low and high *T* regimes align quite well for the Cl^−^ compound as indicated by the dotted line. On the other hand, the CuCl_4_^2−^ and ReO_4_^−^ compounds show perceptibly enhanced conductivity in the smectic mesophase, above the expected levels from the low *T* regime (see dotted lines). The conductivity at 410 K is compared for the Cl^−^ solid and the CuCl_4_^2−^ and ReO_4_^−^ mesophases in Table 4, where the ReO_4_^−^ salt shows the highest value. Due to the enhanced *E*_A_ values in the mesophases for the CuCl_4_^2−^ and ReO_4_^−^ (5.10 eV and 4.61 eV, respectively) and the clear signs of ionic conductivity in the mesophase, we conclude that the mobile charge carriers may be the CuCl_4_^2−^ and ReO_4_^−^ counter-ions here. On the other hand, the reduced *E*_A_ values in the solid phase and lower *T* point towards an electronic contribution. This may imply that the presence of a mesophase is the key factor to enable a high mobility of the CuCl_4_^2−^ and ReO_4_^−^ counter-ions, whereas in the Cl^−^ compound only electronic contributions may prevail due to the absence of a mesophase. All activation energies are summarized in Table 4. Above, the mesophase-liquid transition (Sm-Iso) the *σ*_dc_ *vs.* 1/*T* curves completely change and resemble a metallic trend with decreasing *σ*_dc_ with increasing *T*. We associate this with the complete decomposition of the compounds as mentioned above.

## 3. Materials and Methods

### 3.1. Materials and Physical Measurements

All commercial reagents were used as supplied. Elemental analysis for carbon, hydrogen and nitrogen were carried out by the Microanalytical Service of Complutense University. IR spectra were recorded on a FTIR Thermo Nicolet 200 spectrophotometer in solid state, with samples as KBr pellets, in the 4000–400 cm^−1^ region: *vs.* (very strong), s (strong), m (medium), w (weak), vw (very weak). ^1^H-NMR spectra were performed at room temperature on a Bruker DPX-300 spectrophotometer (NMR Service of Complutense University, Madrid, Spain) from solutions in (CD_3_)_2_CO. Chemical shifts *δ* are listed relative to Me_4_Si using the signal of the deuterated solvent as reference (2.05 ppm), and coupling constants *J* are in hertz. Multiplicities are indicated as s (singlet), d (doublet), t (triplet), m (multiplet), br (broad signal). The ^1^H chemical shifts and coupling constants are accurate to ±0.01 ppm and ±0.3 Hz, respectively. MALDI-TOF-MS analyses were performed in a MALDI-TOF/TOF Bruker model ULTRAFLEX spectrometer at the Mass Spectrometry Service of Complutense University, with dithranol as a matrix.

Phase studies were carried out by polarized light optical microscopy (POM) using an Olympus BX50 microscope equipped with a Linkam THMS 600 heating stage. The temperatures were assigned based on optic observations with polarized light. Measurements of the transition temperatures were made using a Perkin Elmer Pyris 1 differential scanning calorimeter with the sample (1–4 mg) sealed hermetically in aluminum pans and with a heating or cooling rate of 5–10 K·min^−1^. The X-ray diffractograms at variable temperature were recorded on a Panalytical X’Pert PRO MPD diffractometer with Cu-K*α* (1.54 Å) in a *θ-θ* configuration equipped with an Anton Paar HTK1200 heating stage (X-ray Diffraction Service of Complutense University).

The dielectric properties in powder form were probed by alternating current (AC) impedance spectroscopy using an Alpha Analyzer integrated into the Novocontrol BDS 80. Measurements were performed at a frequency (*f*) range of 1 Hz–10 MHz with a 0.1 V applied AC voltage signal, in the temperature (*T*) range of 260 K–480 K (−13 °C–207 °C) upon heating and cooling cycles. Dielectric data were taken under steady state conditions, where the selected *T* was stabilized between 3 and 10 min before taking impedance measurements over the full *f*-range. The temperature increments/reductions for taking impedance measurements were 20 K–2 K steps, where the temperature was increased/decreased in smaller steps near the phase transitions. The compounds in the solid powder state (yellow/green color) were placed between the polished electrodes of a custom-built stainless steel liquid cell with a high surface to thickness ratio [51] as demonstrated in the Supporting Information, Figure S11. The cell was closed with a sapphire plate and placed inside the Novocontrol cryostat.

The dielectric data were obtained at selected temperatures for heating and cooling cycles in terms of the real and imaginary parts (*Z′*, *Z″*) of the complex impedance Z* = *Z′* + i*Z″*. The geometrical factor (*g*) is given by the electrode area divided by electrode distance. Due to experimental limitations, *g* could only be estimated from the weight and density of the powder measured initially, and the measurement cell dimensions. Equivalent circuit fitting of the dielectric data was performed by using commercial Z-View software. The conductivity and permittivity values extracted from the equivalent circuit fits were plotted *vs.* temperature, but only physically meaningful values with sufficiently low fitting errors were considered.

X-ray data collection and structure refinement: Data collection was carried out at room temperature on a Bruker Smart CCD diffractometer using graphite-monochromated Mo-K*α* radiation (*λ* = 0.71073 Å) operating at 50 kV and 35 mA. The data were collected over a hemisphere of the reciprocal space by combination of three exposure sets. Each exposure of 20 s covered 0.3° in *ω*. The cell parameters were determined and refined by a least-squares fit of all reflections. The first 100 frames were recollected at the end of the data collection to monitor crystal decay, and no appreciable decay was observed. A semi-empirical absorption correction was applied. A summary of the fundamental crystal and refinement data is given in Table S2.

The structure was solved by direct methods and refined by full-matrix least-squares procedures on F^2^ [52]. All non-hydrogen atoms were refined anisotropically. The chain carbon atoms, C17-C26, were refined with geometrical restraints and a variable common carbon-carbon distance while the aromatic ring, C9-C14, was also refined using rigid body constraints.

The hydrogen atoms were included in their calculated positions and refined riding on their respective carbon atoms with the exception of H1A, bonded to N1, and H2A, bonded to O2, that have been located in a difference Fourier synthesis and were included and refined riding on their respective linked atoms.

CCDC 1454115 contains the supplementary crystallographic data for this paper. These data can be obtained free of charge from The Cambridge Crystallographic Data Center [53].

### 3.2. Preparation of 2-[3-(4-n-alkyloxyphenyl)propane-1,3-dion-1-yl]pyridinium chloride [HOO^R(n)pyH^]Cl (R = C_6_H_4_OC_n_H_2n+1_, n = 12, 14, 16, 18)

Compounds [HOO^R(n)pyH^][Cl] used as precursors in this work were synthesized according to the procedure previously reported by us [41].

### 3.3. Preparation of 2-[3-(4-n-alkyloxyphenyl)propane-1,3-dion-1-yl]pyridinium salts [HOO^R(n)pyH^][A] (A = BF_4_^−^, ReO_4_^−^, CF_3_SO_3_^−^, NO_3_^−^; R = C_6_H_4_OC_n_H_2n+1_, n = 12, 14, 16, 18)

To a solution of the [HOO^R(n)pyH^][Cl] in 20 mL of dichlorometane, the corresponding salt AgA (A = BF_4_^−^, ReO_4_^−^, CF_3_SO_3_^−^, NO_3_^−^) in 8:2 mL of CH_2_Cl_2_-CH_3_CN and in a 1:1 molar ratio was added under a nitrogen atmosphere. The mixture was stirred for 72 h in the absence of light and then filtered through Celite^®^. The filtrate was concentrated *in vacuo* until a solid precipitated. The yellow solid was filtered and dried *in vacuo*. All compounds were characterized by IR and ^1^H-NMR spectroscopies and CHN or CHNS analyses (deposited as supplementary material). A specific example of the characterization for a compound of each family is given below.

[HOO^R(12)pyH^][BF_4_] (**1**): yellow solid (46%). Found: C, 61.9; H, 6.9; N, 2.9%. C_26_H_36_BF_4_NO_3_·0.1 CH_2_Cl_2_ requires C, 62.0; H, 7.2; N, 2.8%. *ν*_max_(KBr)/cm^−1^: 3268w (NH), 1584s, 1536s, 1514s *ν*(CC + CO), 1073s *ν*(B-F). *δ*_H_ (300 MHz; (CD_3_)_2_CO; Me_4_Si): 0.87 (3 H, t, ^3^*J*_H–H_ 6.1, CH_3_), 1.29 (18 H, m, CH_2_), 1.83 (2 H, m, CH_2_), 4.16 (2 H, t, ^3^*J*_H–H_ 6.5, OCH_2_), 5.62 (CH_2_Cl_2_), 7.15 (2 H, d, ^3^*J*_H–H_ 9.0, H_m_), 7.70 (1 H, s, CH), 8.20 (2 H, d, ^3^*J*_H–H_ 9.0, H_o_), 8.28 (1 H, t, ^3^*J*_H–H_ 6.7, H5), 8.77 (1 H, t, ^3^*J*_H–H_ 7.9, H4), 8.91 (1 H, d, ^3^*J*_H–H_ 8.1, H3), 9.16 (1 H, d, ^3^*J*_H–H_ 5.6, H6)

[HOO^R(12)pyH^][ReO_4_] (**5**): yellow solid (41%). Found: C, 47.1; H, 5.1; N, 2.2%. C_26_H_36_NO_3_ReO_4_ requires: C, 47.3; H, 5.5; N, 2.1%. *ν*_max_(KBr)/cm^−1^: 3087w (NH), 1584s, 1535s, 1510s (CC + CO), 910s (ReO). *δ*_H_ (300 MHz; (CD_3_)_2_CO; Me_4_Si): 0.87 (3 H, t, ^3^*J*_H–H_ 5.7, CH_3_), 1.29 (18 H, m, CH_2_), 1.83 (2 H, m, CH_2_), 4.17 (2 H, t, ^3^*J*_H–H_ 6.5, OCH_2_), 7.16 (2 H, d, ^3^*J*_H–H_ 8.4, H_m_), 7.73 (1 H, s, CH), 8.23 (2 H, d, ^3^*J*_H–H_ 8.5, H_o_), 8.41 (1 H, t, ^3^*J*_H–H_ 6.7, H5), 8.94 (1 H, t, ^3^*J*_H–H_ 7.9, H4), 9.05 (1 H, d, ^3^*J*_H–H_ 8.1, H3), 9.25 (1 H, d, ^3^*J*_H–H_ 5.7, H6).

[HOO^R(12)pyH^][CF_3_SO_3_] (**9**): yellow solid (37%). Found: C, 57.5; H, 6.4; N, 2.8; S, 5.7%. C_26_H_36_NO_3_CF_3_SO_3_ requires C, 57.9; H, 6.5; N, 2.5; S, 5.7%. *ν*_max_(KBr)/cm^−1^: 3100w (NH), 1602s (CC + CO), 1256s, 1032s (SO). *δ*_H_ (300 MHz; (CD_3_)_2_CO; Me_4_Si): 0.83 (3 H, t, ^3^*J*_H–H_ 5.9, CH_3_), 1.35 (18 H, m, CH_2_), 1.79 (2 H, m, CH_2_), 4.10 (2 H, t, ^3^*J*_H–H_ 6.5, OCH_2_), 7.17 (2 H, d, ^3^*J*_H–H_ 8.9, H_m_), 7.73 (1 H, s, CH), 8.23 (2 H, d, ^3^*J*_H–H_ 8.9, H_o_), 8.39 (1 H, t, ^3^*J*_H–H_ 6.0, H5), 8.91 (1 H, t, ^3^*J*_H–H_ 7.2, H4), 9.02 (1 H, d, ^3^*J*_H–H_ 8.3, H3), 9.25 (1 H, d, ^3^*J*_H–H_ 5.5, H6).

[HOO^R(12)pyH^][NO_3_] (**13**): yellow solid (42%). Found: C, 64.4; H, 7.3; N, 5.2%. C_26_H_36_N_2_O_6_·0.2 CH_2_Cl_2_ requires: C, 64.3; H, 7.5; N, 5.7%. *ν*_max_(KBr)/cm^−1^: 3105w (NH), 1583s, 1541s, 1511s (CC + CO), 1398s (NO). *δ*_H_ (300 MHz; (CD_3_)_2_CO; Me_4_Si): 0.83 (3 H, t, ^3^*J*_H–H_ 6.6, CH_3_), 1.35 (18 H, m, CH_2_), 1.79 (2 H, m, CH_2_), 4.10 (2 H, t, ^3^*J*_H–H_ 6.5, OCH_2_), 5.62 (CH_2_Cl_2_), 7.07 (2 H, d, ^3^*J*_H–H_ 8.8, H_m_), 7.57 (1 H, s, CH), 7.65 (1 H, t, ^3^*J*_H–H_ 7.6, H5), 8.05 (1 H, m, H4), 8.08 (2 H, d, ^3^*J*_H–H_ 8.7, H_o_), 8.20 (1 H, d, ^3^*J*_H–H_ 7.9, H3), 8.76 (1 H, d, ^3^*J*_H–H_ 5.3, H6).

### 3.4. Preparation of 2-[3-(4-n-alkyloxyphenyl)propane-1,3-dion-1-yl]pyridinium tetrachlorocuprate(II) [HOO^R(n)pyH^]_2_[CuCl_4_] (R = C_6_H_4_OC_n_H_2n+1_, n = 12, 18)

To a solution of 0.3 mmol of the corresponding [HOO^R(n)pyH^][Cl] in 30 mL of hot acetone was added 0.15 mmol of CuCl_2_·2H_2_O in 5 mL of methanol. The mixture reaction was stirred for 24 h at room temperature. The green precipitate was filtered off, washed with methanol and dried *in vacuo*. Both compounds were characterized by elemental analysis and IR spectroscopic techniques.

[HOO^R(12)pyH^]_2_[CuCl_4_] (**17**): green solid (69%). Found: C, 59.9; H, 6.9; N, 2.7%. C_52_H_72_N_2_O_6_CuCl_4_·H_2_O requires: C, 59.8; H, 7.1; N, 2.7%. *ν*_max_(KBr)/cm^−1^: 3076w (NH), 1600s (CC + CO)

[HOO^R(18)pyH^]_2_[CuCl_4_] (**18**): green solid (76%). Found: C, 63.2; H, 7.6; N, 2.4%. C_64_H_96_N_2_O_6_CuCl_4_·H_2_O requires: C, 63.4; H, 8.1; N, 2.3%. *ν*_max_(KBr)/cm^−1^: 3076w (NH), 1604s (CC + CO)

## 4. Conclusions

The thermotropic study of the five families of salts [HOO^R(n)pyH^]_m_[A] (*n* = 12, 14, 16, 18 and *m* = 1: A = BF_4_^−^, ReO_4_^−^, CF_3_SO_3_^−^, NO_3_^−^; *n* = 12, 18 and *m* = 2: A = CuCl_4_^2−^) has shown as a main result that these compounds exhibit a rich crystalline polymorphic and mesomorphic behavior, where in the latter the counter-ion was found to be the determining factor in the mesophase type (SmA or SmC).

The role of the counter-ion in the smectic mesophases can be summarized as follows:

(a) The presence of the tetrahedral counter-ions BF_4_^−^ or ReO_4_^−^ did not substantially modify neither the melting points nor the mesophase stability range. However the latter was increased when NO_3_^−^ was present as the counter-ion in the salt.
(b) The bulkiest counter-anion CF_3_SO_3_^−^ causes an unfavorable increase in the melting point as well as a significant narrowing of the mesophase range.
(c) The modification on the chain length has no significant influence on the mesomorphic properties in the series (**I**–**IV**). In contrast, the chain length is determinant in compounds of the family **V**, where increasing the alkyl chains from 12 to 18 carbon atoms results in the presence of two mesophases.

From all of these observations, we suggest that the propensity to achieve the best mesomorphism is in the order: CuCl_4_^2−^ > ReO_4_^−^ ≈ BF_4_^−^ > NO_3_^−^ >> CF_3_SO_3_^−^.

In terms of the crystalline polymorphism, we can confirm that the ionic interactions appear to be important when looking for increasing the number of solid phases.

The Cl^−^, CuCl_4_^2−^ and ReO_4_^−^ salts with chain length of 12 carbon atoms have been investigated in detail in terms of their conductivities. We find that the presence of a smectic mesophase in the CuCl_4_^2−^ and ReO_4_^−^ compounds is the key factor to enable sufficiently high mobility of the counter-ions to result in perceptible ionic conductivity, where the ReO_4_^−^ compound exhibits the highest conductivity with a high activation energy of *E*_A_ ≈ 4.6 eV. In the absence of a smectic mesophase in the Cl^−^ compound, the conductivity is dominated by electronic contributions which also dominate the solid phases of the CuCl_4_^2−^ and ReO_4_^−^ compounds.

## Figures and Tables

**Table 1:** Nomenclature proposed for the salts described in this work, including the numbering.

Family	I	II	III	IV	V
*n*/A	BF_4_^−^	ReO_4_^−^	CF_3_SO_3_^−^	NO_3_^−^	CuCl_4_^2−^
12	**BF_4_-12 (1)**	**ReO_4_-12 (5)**	**CF_3_SO_3_-12 (9)**	**NO_3_-12 (13)**	**CuCl_4_-12 (17)**
14	**BF_4_-14 (2)**	**ReO_4_-14 (6)**	**CF_3_SO_3_-14 (10)**	**NO_3_-14 (14)**	**–**
16	**BF_4_-16 (3)**	**ReO_4_-16 (7)**	**CF_3_SO_3_-16 (11)**	**NO_3_-16 (15)**	**–**
18	**BF_4_-18 (4)**	**ReO_4_-18 (8)**	**CF_3_SO_3_-18 (12)**	**NO_3_-18 (16)**	**CuCl_4_-18 (18)**

**Table 2:** Thermal data of compounds of the families **I** (BF_4_-n), **II** (ReO_4_-n), **III** (CF_3_SO_3_-n), **IV** (NO_3_-n) and **V** (CuCl_4_-n).

Compound	Transition ^1^	T^2^/°C	ΔH/kJmol^−1^	Compound	Transition ^1^	T^2^/°C	ΔH/kJmol^−1^
**BF_4_-12 (1)**	Cr→Cr′	99	2.0	**CF_3_SO_3_-12 (9)**	Cr→Cr′	81	14.5
	Cr′→SmA	132	25.0		Cr′→Cr″	98	2.2
	SmA→Iso	163 ^3^	–		Cr″→SmA→Iso	165	18.7 ^4^
**BF_4_-14 (2)**	Cr→Cr′	72	14.1		Iso→SmA→Cr′	142	−19.3 ^4^
	Cr′→Cr″	106	11.9		Cr′→Cr	95	−1.8
	Cr″→SmA	136	26.4	**CF_3_SO_3_-14 (10)**	Cr→Cr′	116	8.8
	SmA→Iso	170 ^3^	–		Cr′→SmA→Iso	169	21.6 ^4^
**BF_4_-16 (3)**	Cr→Cr′	83	10.2		Iso→SmA	156	−1.0
	Cr′→SmA	137	35.7		SmA→Cr′	142	−19.5
	SmA→Iso	176 ^3^	–		Cr′→Cr	112	−7.0
**BF_4_-18 (4)**	Cr→Cr′	85	55.0 ^4^	**CF_3_SO_3_-16 (11)**	Cr→Cr′	79	1.5
	Cr′→Cr″	117			Cr′→Cr″	117	14.1
	Cr″→SmA	133			Cr″→SmA	155	14.1
	SmA→Iso	175 ^3^	–		SmA→Iso	173	0.2
**ReO_4_-12 (5)**	Cr→Cr′	106	13.5		Iso→SmA	170	−0.8
	Cr′→Cr″	114	7.9		SmA→Cr′	129	−15.7
	Cr″→SmA	138	17.7		Cr′→Cr	110	−11.4
	SmA→Iso	173	1.6	**CF_3_SO_3_-18 (12)**	Cr→Cr′	84	5.0
**ReO_4_-14 (6)**	Cr→Cr′	105	26.2 ^4^		Cr′→Cr″	116	13.4
	Cr′→Cr″	116			Cr″→SmA	150	11.2
	Cr″→SmA	133	34.7		SmA→Iso	192	0.2
	SmA→Iso	186	0.5		Iso→SmA	166	−0.5
**ReO_4_-16 (7)**	Cr→Cr′	111	29.6		SmA→Cr	111	−27.0
	Cr′→SmA	135	18.2	**NO_3_-12 (13)**	Cr→SmA	124	41.6 ^4^
	SmA→Iso	178	0.8		SmA→Iso	132	
**ReO_4_-18 (8)**	Cr→Cr′	77	2.8	**NO_3_-14 (14)**	Cr→SmA→Iso	135	48.6 ^4^
	Cr′→Cr″	107	22.4	**NO_3_-16 (15)**	Cr→SmA→Iso	136	56.4 ^4^
	Cr″→SmA	127	5.4	**NO_3_-18 (16)**	Cr→SmA→Iso	135	50.1 ^4^
	SmA→Iso	182	0.2	**CuCl_4_-12 (17)**	Cr→SmC	110	32.8
					SmC→Iso	185	54.6
				**CuCl_4_-18 (18)**	Cr→Cr′	79	73.1 ^4^
					Cr′→SmC	92	
					SmC→Cr″	108	−34.2
					Cr″→SmC	174	42.9
					SmC→Iso	199	45.7

^1^ Cr, Cr′, Cr″ = solid phases, SmA = smectic A mesophase, SmC = smectic C mesophase, Iso = isotropic liquid; ^2^ DSC onset peaks given in the first heating process; ^3^ Detected by POM; ^4^ Overlapped processes.

**Table 3:** X-ray diffraction data of selected compounds.

Compound	T/°C	2*θ*/°	*d*_obs_^1^/Å	*d*_cal_^1^/Å	[*hkl*] ^2^	Lattice Parameter/Å
**BF_4_-16 (3)**	130	2.5	34.5	34.5	(001)	*c* = 34.5
		5.0	17.5	17.3	(002)	
		7.5	11.7	11.5	(003)	
		19	5.2	–	halo	
**ReO_4_-16 (7)**	140	1.8	47.0	47.0	(001)	*c* = 47.0
		3.6	24.5	23.5	(002)	
		20	4.9	–	halo	
**CF_3_SO_3_-18 (12)**	160	2.8	31.5	31.5	(001)	*c* = 31.5
		5.6	15.7	15.7	(002)	
		18	5.4	–	halo	
**NO_3_-12 (13)**	120	3.2	27.1	27.1	(001)	*c* = 27.1
		6.3	13.9	13.6	(002)	
		18.5	5.3	–	halo	
**CuCl_4_-18 (18)**	103	3.3	26.5	26.5	(001)	*c = 26.5*
		6.8	13.0	13.2	(002)	
		10.2	8.7	8.8	(003)	
		18.5	5.3	–	halo	

^1^ *d*_obs_ and *d*_cal_ are the observed and calculated diffraction spacings; ^2^ [*hkl*] are the Miller indices of the reflections.

**Table 4:** Activation energies *E_A_* for the Cl^−^, CuCl_4_^2−^ and ReO_4_^−^ salts in the solid phase and the mesophase. Note that the Cl^−^ does not exhibit a mesophase. Conductivity values are given at 410 K in the CuCl_4_^2−^ and ReO_4_^−^ mesophases and the solid phase for the Cl^−^.

[A]	*E_A_* (Solid)	*E_A_* (Mesophase)	*σ*_410K_ (Ωcm)^−1^
Cl^−^	3.08/3.14	–	6.3 × 10^−7^
CuCl_4_^2−^	2.20	5.10	8.7 × 10^−8^
ReO_4_^−^	2.71	4.61	8.7 × 10^−6^

## Supplementary Materials

The following are available online at www.mdpi.com/1996-1944/9/5/360/s1.

## Abbreviations

The following abbreviations are used in this manuscript:

IL	Ionic Liquid
ILC	Ionic Liquid Crystal
LC	Liquid Crystal
Iso	Isotropic liquid
Cr, Cr′, Cr″	Solid Phases
Sm	Smectic Mesophase
POM	Polarized light Optical Microscopy
DSC	Differential Scanning Calorimetry
XRD	X-ray Diffraction
CPE	Constant Phase Element
RC	Resistor-Capacitor
NMR	Nuclear Magnetic Resonance
