# Supplementary material for: Diketonylpyridinium Cations as a Support of New Ionic Liquid Crystals and Ion-Conductive Materials: Analysis of Counter-Ion Effects
Source: Materials (Basel). 2016 May 12;9(5):360. doi: 10.3390/ma9050360 (PMC5503038; doi:10.3390/ma9050360)
Supplement: Supplementary file 1 [file materials-09-00360-s001.pdf]

Supplementary Material: Diketonylpyridinium Cations as a Support of New Ionic Liquid Crystals and Ion-Conductive Materials: Analysis of Counterion Effects

María Jesús Pastor, Cristián Cuerva, José A. Campo, Rainer Schmidt, María Rosario Torres and Mercedes Cano

Characterization of Compounds [HOO$^{R(n)pyH}$][A] (A = BF_4^-, ReO_4^-, $CF_3SO_3^-$, NO_3^-) by Elemental Analysis (CHN or CHNS), IR and ^{1}H-NMR

[HOO$^{R(14)pyH}$][BF_4] (**2**): yellow solid (49%). Found: C, 59.9; H, 6.8; N, 2.5%. $C_{28}H_{40}BF_4NO_3$·0.5 CH_2Cl_2 requires C, 60.2; H, 7.2; N, 2.5%. ν_{max}(KBr)/cm^{-1}: 3255w (NH), 1564s, 1529s, 1507s (CC + CO), 1056s (B-F). δ_H (300 MHz; $(CD_3)_2CO$; Me_4Si): 0.83 (3 H, t, $^3J_{H-H}$ 6.4, CH_3), 1.24 (22 H, m, CH_2), 1.83 (2 H, m, CH_2), 4.16 (2 H, t, $^3J_{H-H}$ 6.5, OCH_2), 5.62 (CH_2Cl_2), 7.14 (2 H, d, $^3J_{H-H}$ 9.0, H_m), 7.71 (1 H, s, CH), 8.22 (2 H, d, $^3J_{H-H}$ 8.9, H_o), 8.39 (1 H, t, $^3J_{H-H}$ 6.8, H5), 8.90 (1 H, t, $^3J_{H-H}$ 7.8, H4), 9.03 (1 H, d, $^3J_{H-H}$ 8.1, H3), 9.22 (1 H, d, $^3J_{H-H}$ 5.6, H6)

[HOO$^{R(16)pyH}$][BF_4] (**3**): yellow solid (50%). Found: C, 64.6; H, 7.8; N, 2.6%. $C_{30}H_{44}BF_4NO_3$ requires C, 65.1; H, 8.0; N, 2.5%. ν_{max}(KBr)/cm^{-1}: 3250w (NH), 1605s, 1568s, 1533s (CC + CO), 1083s (B-F). δ_H (300 MHz; $(CD_3)_2CO$; Me_4Si): 0.86 (3 H, t, $^3J_{H-H}$ 6.5, CH_3), 1.27 (26 H, m, CH_2), 1.85 (2 H, m, CH_2), 4.15 (2 H, t, $^3J_{H-H}$ 6.5, OCH_2), 7.14 (2 H, d, $^3J_{H-H}$ 9.0, H_m), 7.67 (1 H, s, CH), 8.17 (2 H, d, $^3J_{H-H}$ 9.0, H_o), 8.16 (1 H, m, H5), 8.62 (1 H, t, $^3J_{H-H}$ 7.8, H4), 8.75 (1 H, d, $^3J_{H-H}$ 8.1, H3), 9.07 (1 H, d, $^3J_{H-H}$ 5.5, H6)

[HOO$^{R(18)pyH}$][BF_4] (**4**): yellow solid (53%). Found: C, 65.2; H, 8.0; N, 2.4%. $C_{32}H_{48}BF_4NO_3$·0.1 CH_2Cl_2 requires C, 65.3; H, 8.2; N, 2.4%. ν_{max}(KBr)/cm^{-1}: 3251w (NH), 1605s, 1570s, 1534s (CC + CO), 1084s (B-F). δ_H (300 MHz; $(CD_3)_2CO$; Me_4Si): 0.87 (3 H, t, $^3J_{H-H}$ 6.0, CH_3), 1.28 (30 H, m, CH_2), 1.85 (2 H, m, CH_2), 4.17 (2 H, t, $^3J_{H-H}$ 6.4, OCH_2), 5.62 (CH_2Cl_2), 7.15 (2 H, d, $^3J_{H-H}$ 8.8, H_m), 7.72 (1 H, s, CH), 8.19 (2 H, d, $^3J_{H-H}$ 9.0, H_o), 8.25 (1 H, m, H5), 8.69 (1 H, t, $^3J_{H-H}$ 8.1, H4), 8.85 (1 H, d, $^3J_{H-H}$ 8.3, H3), 9.13 (1 H, d, $^3J_{H-H}$ 5.6, H6)

[HOO$^{R(14)pyH}$][ReO_4] (**6**): yellow solid (53%). Found: C, 48.2; H, 5.7; N, 2.1%. $C_{28}H_{40}NO_3ReO_4$ requires: C, 48.0; H, 5.8; N, 2.0%. ν_{max}(KBr)/cm^{-1}: 3099w (NH), 1587s, 1535s, 1515s (CC + CO), 912s (ReO). δ_H (300 MHz; $(CD_3)_2CO$; Me_4Si): 0.87 (3 H, t, $^3J_{H-H}$ 6.4, CH_3), 1.28 (22 H, m, CH_2), 1.83 (2 H, m, CH_2), 4.17 (2 H, t, $^3J_{H-H}$ 6.5, OCH_2), 7.16 (2 H, d, $^3J_{H-H}$ 8.4, H_m), 7.73 (1 H, s, CH), 8.23 (2 H, d, $^3J_{H-H}$ 8.4, H_o), 8.45 (1 H, t, $^3J_{H-H}$ 6.7, H5), 8.98 (1 H, t, $^3J_{H-H}$ 7.8, H4), 9.10 (1 H, d, $^3J_{H-H}$ 8.1, H3), 9.28 (1 H, d, $^3J_{H-H}$ 5.6, H6)

[HOO$^{R(16)pyH}$][ReO_4] (**7**): yellow solid (48%). Found: C, 50.1; H, 6.0; N, 1.9%. $C_{30}H_{44}NO_3ReO_4$ requires: C, 50.3; H, 6.2; N, 1.9%. ν_{max}(KBr)/cm^{-1}: 3098w (NH), 1586s, 1536s, 1514s (CC + CO), 912s (ReO). δ_H (300 MHz; $(CD_3)_2CO$; Me_4Si): 0.87 (3 H, t, $^3J_{H-H}$ 5.9, CH_3), 1.28 (26 H, m, CH_2), 1.83 (2 H, m, CH_2), 4.17 (2 H, t, $^3J_{H-H}$ 6.5, OCH_2), 7.16 (2 H, d, $^3J_{H-H}$ 9.0, H_m), 7.74 (1 H, s, CH), 8.24 (2 H, d, $^3J_{H-H}$ 8.8, H_o), 8.49 (1 H, t, $^3J_{H-H}$ 6.7, H5), 9.02 (1 H, t, $^3J_{H-H}$ 7.6, H4), 9.13 (1 H, d, $^3J_{H-H}$ 8.1, H3), 9.29 (1 H, d, $^3J_{H-H}$ 5.7, H6)

[HOO$^{R(18)pyH}$][ReO_4] (**8**): yellow solid (57%). Found: C, 51.7; H, 6.1; N, 2.9%. $C_{32}H_{48}NO_3ReO_4$·0.6 CH_3CN requires: C, 51.8; H, 6.5; N, 2.9%. ν_{max}(KBr)/cm^{-1}: 3088w (NH), 1588s, 1535s, 1514s (CC + CO), 912s (ReO). δ_H (300 MHz; $(CD_3)_2CO$; Me_4Si): 0.87 (3 H, t, $^3J_{H-H}$ 6.4, CH_3), 1.28 (30 H, m, CH_2), 1.83 (2 H, m, CH_2), 4.17 (2 H, t, $^3J_{H-H}$ 6.4, OCH_2), 7.16 (2 H, d, $^3J_{H-H}$ 8.9, H_m), 7.72 (1 H, s, CH), 8.23 (2 H, d, $^3J_{H-H}$ 8.8, H_o), 7.92 (1 H, m, H5), 8.39 (1 H, m, H4), 9.51 (1 H, m, H3), 8.96 (1 H, d, $^3J_{H-H}$ 5.4, H6)

[HOO$^{R(14)pyH}$][CF_3SO_3] (**10**): yellow solid (38%). Found: C, 58.2; H, 6.5; N, 2.6; S, 5.4%. $C_{28}H_{40}NO_3CF_3SO_3$·0.2 CH_2Cl_2 requires C, 58.1; H, 6.6; N, 2.3; S, 5.3%. ν_{max}(KBr)/cm^{-1}: 3100w (NH), 1603s (CC + CO), 1256s, 1034s (SO). δ_H (300 MHz; $(CD_3)_2CO$; Me_4Si): 0.86 (3 H, t, $^3J_{H-H}$ 6.5, CH_3), 1.35 (22 H, m, CH_2), 1.80 (2 H, m, CH_2), 4.05 (2 H, t, $^3J_{H-H}$ 6.5, OCH_2), 5.62 (CH_2Cl_2), 7.15 (2 H, d, $^3J_{H-H}$ 8.9,

H_m), 7.67 (1 H, s, CH), 8.16 (2 H, d, $^3J_{H–H}$ 8.8, H_o), 8.43 (1 H, m, H5), 8.95 (1 H, m, H4), 9.10 (1 H, m, H3), 9.25 (1 H, d, $^3J_{H–H}$ 5.5, H6)

[$HOO^{R(16)pyH}$][CF_3SO_3] (**11**): yellow solid (45%). Found: C, 60.4; H, 7.1; N, 2.6; S, 5.2%. $C_{30}H_{44}NO_3CF_3SO_3$ requires C, 60.5; H, 7.2; N, 2.3; S, 5.2%. ν_{max}(KBr)/cm^{-1}: 3105w (NH), 1603s (CC + CO), 1256s, 1032s (SO). δ_H (300 MHz; $(CD_3)_2CO$; Me_4Si): 0.91 (3 H, t, $^3J_{H–H}$ 6.3, CH_3), 1.39 (26 H, m, CH_2), 1.86 (2 H, m, CH_2), 4.20 (2 H, t, $^3J_{H–H}$ 6.4, OCH_2), 7.19 (2 H, d, $^3J_{H–H}$ 8.9, H_m), 7.74 (1 H, s, CH), 8.25 (2 H, d, $^3J_{H–H}$ 9.0, H_o), 8.37 (1 H, t, $^3J_{H–H}$ 6.7, H5), 8.87 (1 H, t, $^3J_{H–H}$ 7.8, H4), 9.00 (1 H, d, $^3J_{H–H}$ 7.9, H3), 9.23 (1 H, d, $^3J_{H–H}$ 5.6, H6)

[$HOO^{R(18)pyH}$][CF_3SO_3] (**12**): yellow solid (48%). Found: C, 60.4; H, 7.1; N, 2.5; S, 5.0%. $C_{32}H_{48}NO_3CF_3SO_3{\cdot}0.2\ CH_2Cl_2{\cdot}0.1\ CH_3CN$ requires C, 60.3; H, 7.4; N, 2.3; S, 4.8%. ν_{max}(KBr)/cm^{-1}: 3099w (NH), 1602s (CC + CO), 1256s, 1032s (SO). δ_H (300 MHz; $(CD_3)_2CO$; Me_4Si): 0.91 (3 H, t, $^3J_{H–H}$ 6.4, CH_3), 1.39 (30 H, m, CH_2), 1.86 (2 H, m, CH_2), 4.21 (2 H, t, $^3J_{H–H}$ 6.5, OCH_2), 5.62 (CH_2Cl_2), 7.19 (2 H, d, $^3J_{H–H}$ 9.0, H_m), 7.75 (1 H, s, CH), 8.27 (2 H, d, $^3J_{H–H}$ 9.0, H_o), 8.47 (1 H, t, $^3J_{H–H}$ 6.9, H5), 8.98 (1 H, t, $^3J_{H–H}$ 7.8, H4), 9.10 (1 H, d, $^3J_{H–H}$ 8.0, H3), 9.29 (1 H, d, $^3J_{H–H}$ 5.6, H6)

[$HOO^{R(14)pyH}$][NO_3] (**14**): yellow solid (40%). Found: C, 66.9; H 7.8; N, 5.8%. $C_{28}H_{40}N_2O_6$ requires: C, 67.2; H, 8.0; N, 5.6%. ν_{max}(KBr)/cm^{-1}: 3106w (NH), 1583s, 1540s, 1514s (CC + CO), 1400s (NO). δ_H (300 MHz; $(CD_3)_2CO$; Me_4Si): 0.83 (3 H, t, $^3J_{H–H}$ 6.2, CH_3), 1.35 (22 H, m, CH_2), 1.79 (2 H, m, CH_2), 4.10 (2 H, t, $^3J_{H–H}$ 6.5, OCH_2), 7.12 (2 H, d, $^3J_{H–H}$ 8.9, H_m), 7.60 (1 H, s, CH), 7.63 (1 H, m, H5), 8.05 (1 H, m, H4), 8.09 (2 H, d, $^3J_{H–H}$ 8.9, H_o), 8.17 (1 H, d, $^3J_{H–H}$ 7.8, H3), 8.76 (1 H, d, $^3J_{H–H}$ 5.8, H6)

[$HOO^{R(16)pyH}$][NO_3] (**15**): yellow solid (46%). Found: C, 67.6; H, 8.1; N, 5.4%. $C_{30}H_{44}N_2O_6$ requires: C, 67.3; H, 8.3; N, 5.2%. ν_{max}(KBr)/cm^{-1}: 3106w (NH), 1584s, 1543s, 1514s (CC + CO), 1401s (NO). δ_H (300 MHz; $(CD_3)_2CO$; Me_4Si): 0.90 (3 H, t, $^3J_{H–H}$ 6.4, CH_3), 1.44 (26 H, m, CH_2), 1.85 (2 H, m, CH_2), 4.17 (2 H, t, $^3J_{H–H}$ 6.5, OCH_2), 7.14 (2 H, d, $^3J_{H–H}$ 8.9, H_m), 7.63 (1 H, s, CH), 7.65 (1 H, m, H5), 8.07 (1 H, m, H4), 8.10 (2 H, d, $^3J_{H–H}$ 8.9, H_o), 8.20 (1 H, d, $^3J_{H–H}$ 7.9, H3), 8.79 (1 H, d, $^3J_{H–H}$ 5.5, H6)

[$HOO^{R(18)pyH}$][NO_3] (**16**): yellow solid (48%). Found: C, 68.4; H, 8.4; N, 5.1%. $C_{32}H_{48}N_2O_6{\cdot}0.1\ CH_2Cl_2$ requires: C, 68.2; H, 8.6; N, 5.0%. ν_{max}(KBr)/cm^{-1}: 3105w (NH), 1584s, 1539s, 1514s (CC + CO), 1401s (NO). δ_H (300 MHz; $(CD_3)_2CO$; Me_4Si): 0.90 (3 H, t, $^3J_{H–H}$ 6.1, CH_3), 1.44 (30 H, m, CH_2), 1.85 (2 H, m, CH_2), 4.17 (2 H, t, $^3J_{H–H}$ 6.5, OCH_2), 7.14 (2 H, d, $^3J_{H–H}$ 8.9, H_m), 7.63 (1 H, s, CH), 7.65 (1 H, m, H5), 8.06 (1 H, m, H4), 8.10 (2 H, d, $^3J_{H–H}$ 9.0, H_o), 8.20 (1 H, d, $^3J_{H–H}$ 7.9, H3), 8.78 (1 H, d, $^3J_{H–H}$ 5.5, H6)

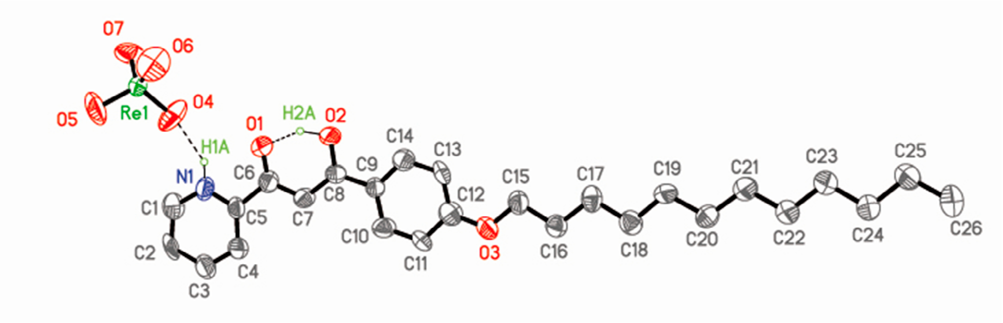

Figure S1. ORTEP plot for [$HOO^{R(12)pyH}$][ReO_4] (**5**) with 40% probability. Hydrogen atoms, except H1A and H2A, have been omitted for clarity.

Table S1. Bond distances (Å) and angles (°) for [HOO$^{R(12)pyH}$][ReO_4] (**5**).

		N(1)–C(1)–C(2)	120(2)
		C(3)–C(2)–C(1)	117(2)
		C(2)–C(3)–C(4)	121(2)
		C(3)–C(4)–C(5)	116(2)
C(1)–N(1)	1.34(2)	N(1)–C(5)–C(4)	123(2)
C(1)–C(2)	1.41(3)	N(1)–C(5)–C(6)	111.9(19)
C(2)–C(3)	1.39(3)	C(4)–C(5)–C(6)	125(2)
C(3)–C(4)	1.41(3)	O(1)–C(6)–C(7)	123.0(19)
C(4)–C(5)	1.40(2)	O(1)–C(6)–C(5)	117.3(19)
C(5)–N(1)	1.29(2)	C(7)–C(6)–C(5)	119.7(19)
C(5)–C(6)	1.51(3)	C(6)–C(7)–C(8)	121.3(19)
C(6)–O(1)	1.22(2)	O(2)–C(8)–C(7)	117.8(17)
C(6)–C(7)	1.39(2)	O(2)–C(8)–C(9)	115.7(16)
C(7)–C(8)	1.43(2)	C(7)–C(8)–C(9)	126.4(18)
C(8)–O(2)	1.30(2)	C(10)–C(9)–C(14)	120.0
C(8)–C(9)	1.46(2)	C(10)–C(9)–C(8)	120.3(12)
C(9)–C(10)	1.3900	C(14)–C(9)–C(8)	119.6(12)
C(9)–C(14)	1.3900	C(11)–C(10)–C(9)	120.0
C(10)–C(11)	1.3900	C(10)–C(11)–C(12)	120.0
C(11)–C(12)	1.3900	O(3)–C(12)–C(11)	117.3(11)
C(12)–O(3)	1.345(15)	O(3)–C(12)–C(13)	122.7(11)
C(12)–C(13)	1.3900	C(11)–C(12)–C(13)	120.0
C(13)–C(14)	1.3900	C(12)–C(13)–C(14)	120.0
C(15)–O(3)	1.40(2)	C(13)–C(14)–C(9)	120.0
C(15)–C(16)	1.50(2)	O(3)–C(15)–C(16)	111.3(17)
C(16)–C(17)	1.51(2)	C(15)–C(16)–C(17)	113.0(16)
C(17)–C(18)	1.518(5)	C(18)–C(17)–C(16)	113.5(15)
C(18)–C(19)	1.519(5)	C(17)–C(18)–C(19)	111.2(14)
C(19)–C(20)	1.517(5)	C(20)–C(19)–C(18)	113.0(13)
C(20)–C(21)	1.518(5)	C(19)–C(20)–C(21)	116.1(13)
C(21)–C(22)	1.517(5)	C(22)–C(21)–C(20)	115.8(13)
C(22)–C(23)	1.518(5)	C(21)–C(22)–C(23)	114.0(13)
C(23)–C(24)	1.520(5)	C(22)–C(23)–C(24)	112.6(13)
C(24)–C(25)	1.519(5)	C(25)–C(24)–C(23)	111.0(14)
C(25)–C(26)	1.520(5)	C(26)–C(25)–C(24)	111.9(16)
O(4)–Re(1)	1.657(14)	C(5)–N(1)–C(1)	121(2)
O(5)–Re(1)	1.622(14)	C(12)–O(3)–C(15)	120.9(15)
O(6)–Re(1)	1.651(15)	O(5)–Re(1)–O(7)	110.2(9)
O(7)–Re(1)	1.650(13)	O(5)–Re(1)–O(4)	113.7(11)
		O(7)–Re(1)–O(4)	105.9(9)
		O(5)–Re(1)–O(6)	106.8(11)
		O(7)–Re(1)–O(6)	110.1(9)
		O(4)–Re(1)–O(6)	110.2(9)

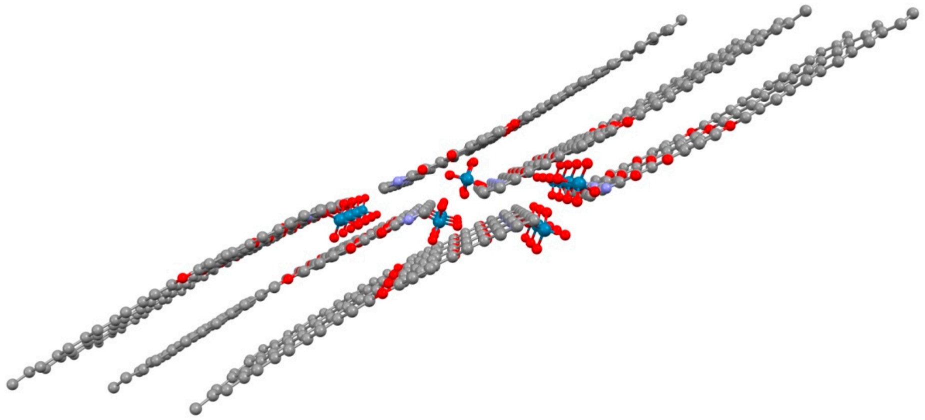

Figure S2. View of the packing through the *b* axis showing the layer-type distribution generated by C–H···O hydrogen-bonds.

Table S2. Crystal and refinement data for [HOO$^{R(12)pyH}$][ReO_4] (**5**).

Empirical formula	[$C_{26}H_{36}NO_7Re$]
Formula weight	660.76
Crystal system	Monoclinic
Space group	$P2_1/c$
Space group number	14
a/Å	20.023(13)
b/Å	9.662(6)
c/Å	15.091(9)
β (°)	109.666(12)
V/Å^3	2749(3)
Z	4
T/K	293(2)
F(000)	1320
ρ_c/g cm^{-3}	1.596
μ/mm^{-1}	4.462
Scan technique	ω and ϕ
Data collected	(−23, −11, −16) to (22, 11, 17)
θ range (°)	1.08 to 25.00
Reflections collected	20417
Independent reflections	4838 (R_{int} = 0.1968)
Completeness to maximum θ (%)	99.8
Data/restraints/parameters	4838/87/304
Observed reflections [$I > 2\sigma(I)$]	1642
R [1]	0.0704
R_{wF} [2]	0.2202

[1] $\Sigma[|F_o| - |F_c|]/\Sigma[|F_o|]$; [2] $\{\Sigma[w(F_o^2 - F_c^2)^2]/\Sigma[w(F_o^2)^2]\}^{1/2}$.

Table S3. Hydrogen bond geometries (lengths in Å and angles in degrees).

D–H···A	d(D–H)	d(H···A)	d(D···A)	<(D–H···A)
N1–H1A···O4	0.930	1.929	2.749(2)	145.9
C2–H2···O7 [1]	0.930	2.560	3.218(2)	128.1
C1–H1···O7 [2]	0.930	2.617	3.330(2)	133.8
C4–H4···O5 [3]	0.930	2.461	3.272(2)	145.8

Symmetry operations: [1] $x, y - 1, z$; [2] $-x, -y + 1, -z + 2$; [3] $x, -y + 1/2, z - ½$.

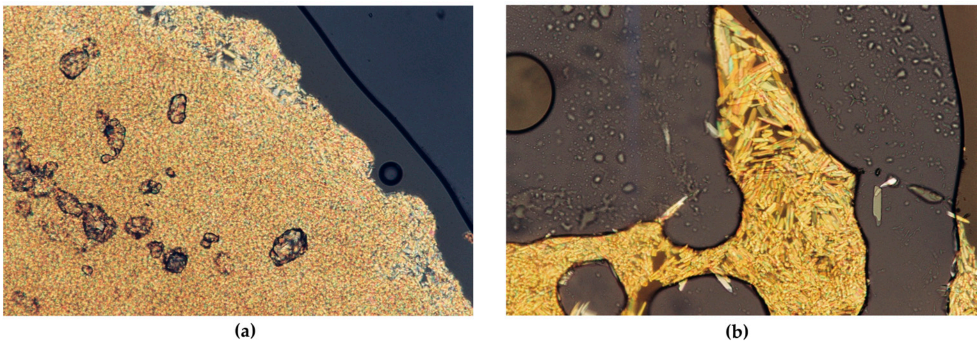

Figure S3. POM photomicrographs of: (**a**) **NO3-14** at 117 °C on cooling; (**b**) **NO3-18** at 121 °C on cooling.

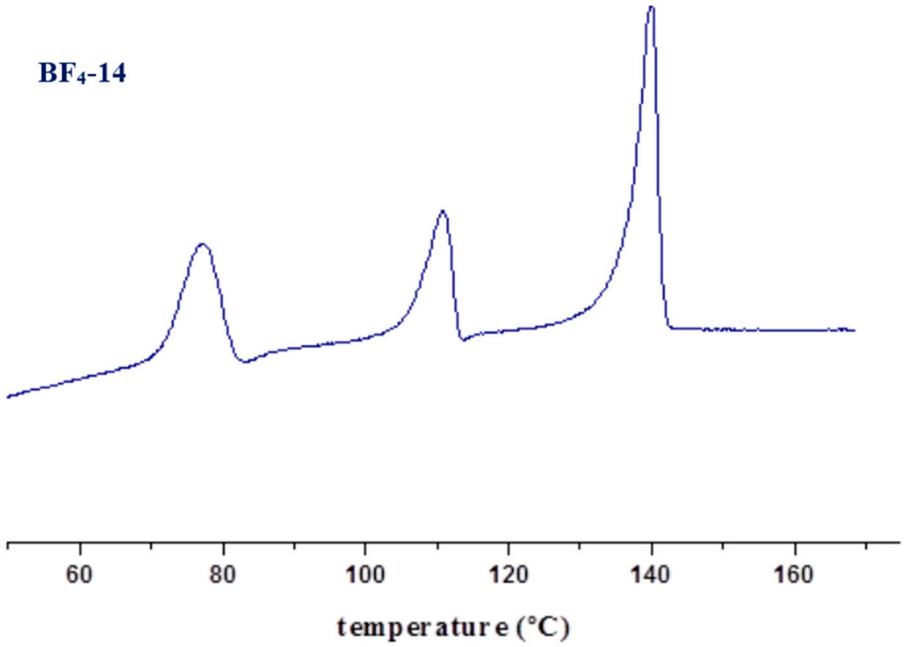

Figure S4. DSC trace of compound **2** in the first heating (endothermic up).

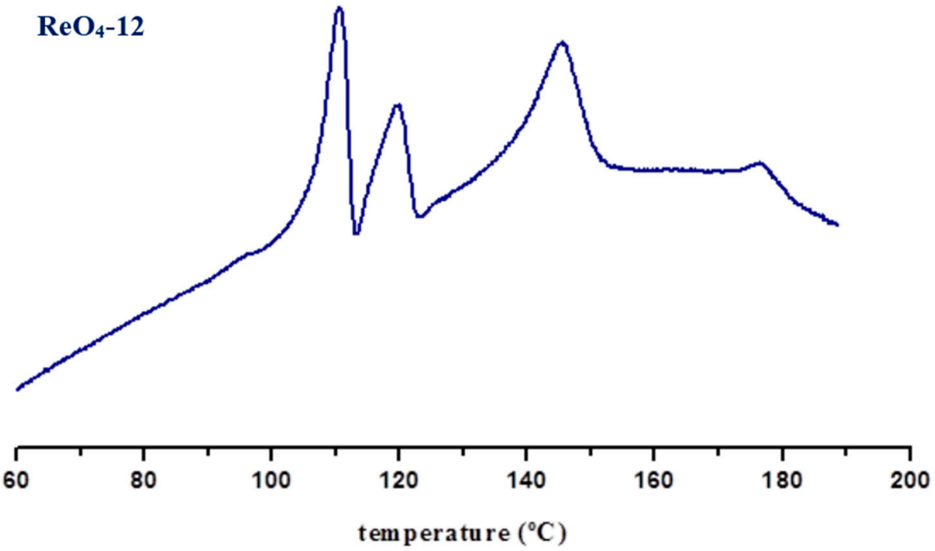

Figure S5. DSC trace of compound **5** in the first heating (endothermic up).

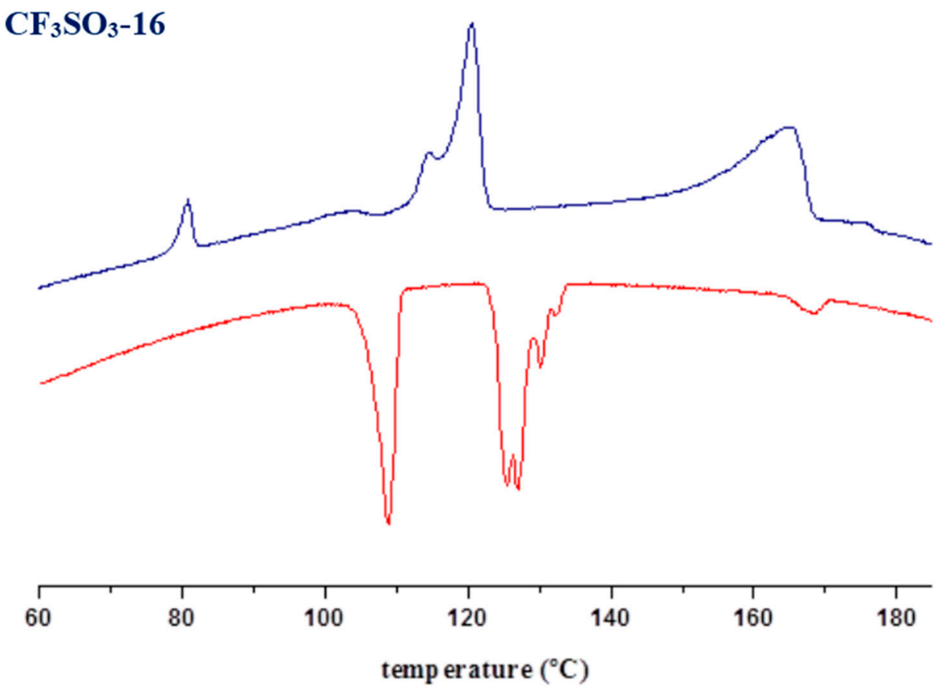

Figure S6. DSC trace of compound **11** in the first heating (blue line)/cooling (red line) cycle (endothermic up).

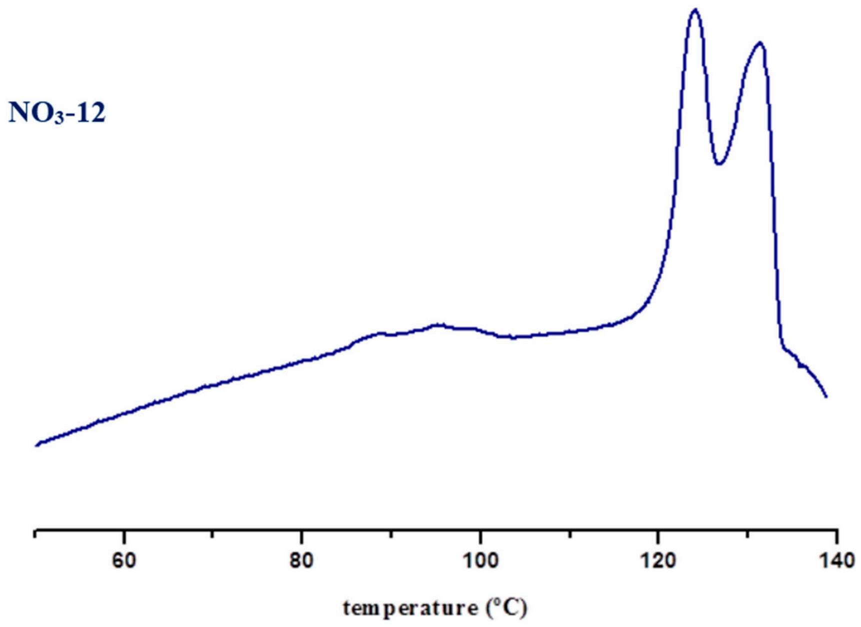

Figure S7. DSC trace of compound **13** in the first heating (endothermic up).

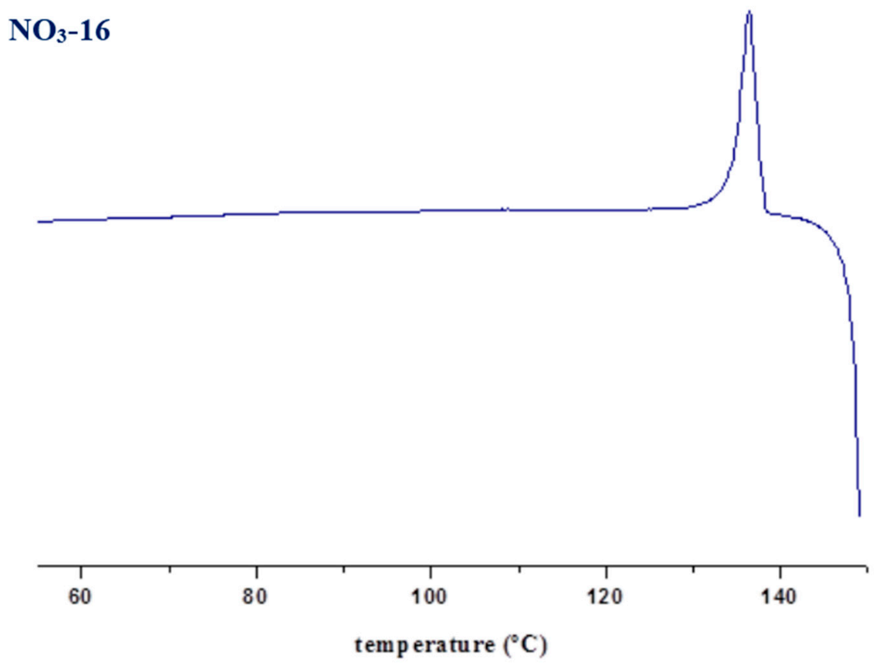

Figure S8. DSC trace of compound **15** in the first heating (endothermic up).

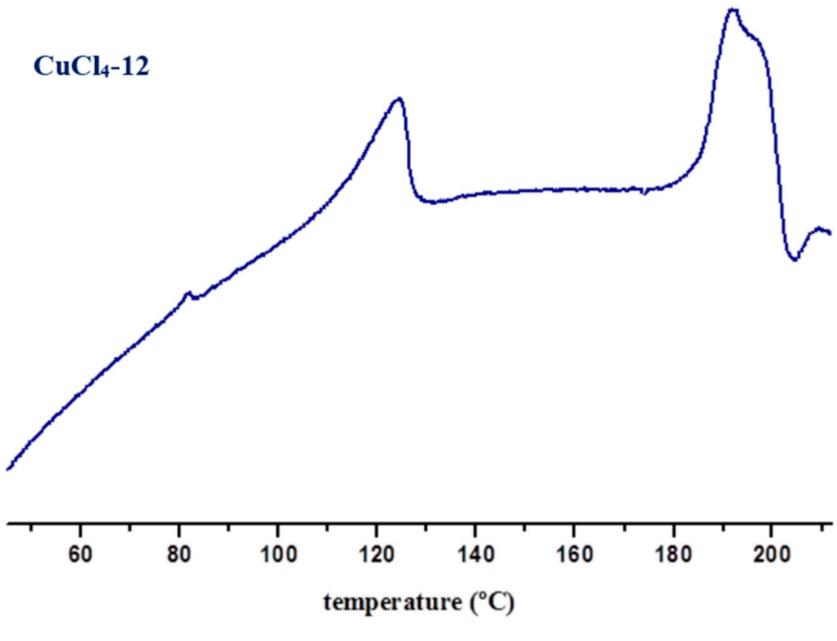

Figure S9. DSC trace of compound **17** in the first heating (endothermic up).

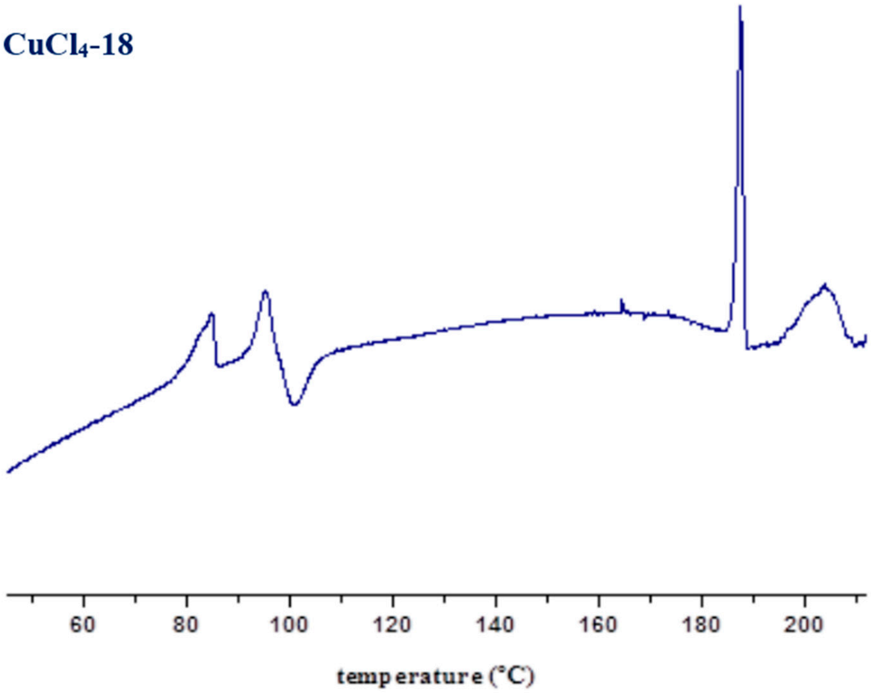

Figure S10. DSC trace of compound **18** in the first heating (endothermic up).

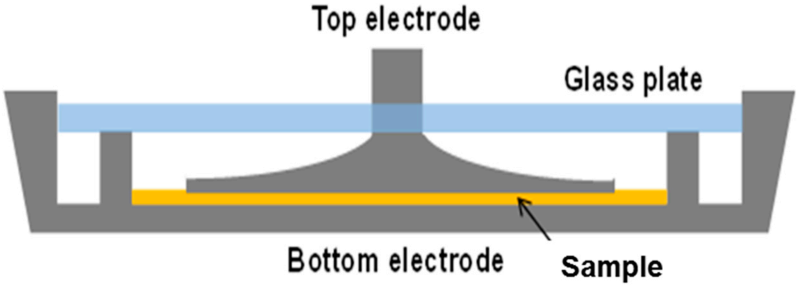

Figure S11. Custom-built liquid-solid measurement cell to obtain the conductivity and dielectric properties by impedance spectroscopy in the powder and liquid-crystalline state between the top and bottom electrodes.
